# RNA-Seq of *Bacillus licheniformis*: active regulatory RNA features expressed within a productive fermentation

**DOI:** 10.1186/1471-2164-14-667

**Published:** 2013-10-01

**Authors:** Sandra Wiegand, Sascha Dietrich, Robert Hertel, Johannes Bongaerts, Stefan Evers, Sonja Volland, Rolf Daniel, Heiko Liesegang

**Affiliations:** 1 Department of Genomic and Applied Microbiology & Göttingen Genomics Laboratory, Institut für Mikrobiologie und Genetik, Norddeutsches Zentrum für Mikrobielle Genomforschung, Georg-August-Universität Göttingen, Grisebachstr. 8, D-37077 Göttingen, Germany; 2 Henkel AG & Co. KGaA, Henkelstraße 67, D-40191 Düsseldorf, Germany

**Keywords:** dRNA-Seq, RNA-based regulation, UTR, ncRNA, sRNA, Antisense RNA, Subtilisin, Transcription start site, Operon prediction, Reannotation

## Abstract

**Background:**

The production of enzymes by an industrial strain requires a complex adaption of the bacterial metabolism to the conditions within the fermenter. Regulatory events within the process result in a dynamic change of the transcriptional activity of the genome. This complex network of genes is orchestrated by proteins as well as regulatory RNA elements. Here we present an RNA-Seq based study considering selected phases of an industry-oriented fermentation of *Bacillus licheniformis*.

**Results:**

A detailed analysis of 20 strand-specific RNA-Seq datasets revealed a multitude of transcriptionally active genomic regions. 3314 RNA features encoded by such active loci have been identified and sorted into ten functional classes. The identified sequences include the expected RNA features like housekeeping sRNAs, metabolic riboswitches and RNA switches well known from studies on *Bacillus subtilis* as well as a multitude of completely new candidates for regulatory RNAs. An unexpectedly high number of 855 RNA features are encoded antisense to annotated protein and RNA genes, in addition to 461 independently transcribed small RNAs. These antisense transcripts contain molecules with a remarkable size range variation from 38 to 6348 base pairs in length. The genome of the type strain *B. licheniformis* DSM13 was completely reannotated using data obtained from RNA-Seq analyses and from public databases.

**Conclusion:**

The hereby generated data-sets represent a solid amount of knowledge on the dynamic transcriptional activities during the investigated fermentation stages. The identified regulatory elements enable research on the understanding and the optimization of crucial metabolic activities during a productive fermentation of *Bacillus licheniformis* strains.

## Background

*Bacillus licheniformis* is a spore-forming soil bacterium closely related to the Gram-positive model organism *Bacillus subtilis.* The species’ saprophytic life style, based on the secretion of biopolymer-degrading enzymes, predestinates strains of *B. licheniformis* as ideal candidates for the large-scale industrial production of exoenzymes, such as amylases and peptide antibiotics [1]. Especially its high capacity of secreting overexpressed alkaline serine proteases has made *B. licheniformis* one of the most important bacterial workhorses in industrial enzyme production [2]. Due to their high stability and relatively low substrate specificity, alkaline serine proteases like subtilisins are crucial additives to household detergents and the greatest share on the worldwide enzyme market [2,3]. Attempts to optimize the productivity have addressed the fermentation process [4,5], protein-engineering [3,6,7], and cellular influences on protein quality and quantity [2,8]. Since the 4.2 Mb circular genome of the type strain *B. licheniformis* DSM13 was published in 2004 [1,9], several genome-based studies targeting strain improvement have been performed successfully [10,11]. However, genome-based studies are limited to information directly accessible from the DNA sequence and cannot benefit from knowledge of the active transcriptome. Considering that the regulatory network represented by protein- and RNA-based regulators determines the performance of an industrial-oriented fermentation process [12] RNA-Seq data might contribute to further optimization approaches.

RNA-based regulatory elements are involved in the regulation of metabolism, growth processes, the adaptation to stress and varying culture conditions [13] and can be divided into two main categories. The first category comprises non-coding RNAs (ncRNAs). *Trans*-encoded ncRNAs, often referred to as small RNAs (sRNAs), are encoded independently from protein genes and are able to modulate the mRNA of genes located at distant chromosomal loci or to interact with target proteins [14]. Upon formation of secondary structures, *trans*-encoded ncRNAs interact with their target RNAs by imperfect base pairing, which is triggered by the binding of a seed region of at least six contiguous nucleotides [15,16]. This mechanism allows the sRNA to address multiple targets, thus orchestrating different members of one regulon [14,17]. It has been shown that sRNAs affect mRNA degradation and translation and modulate protein activity [14,16]. A second class of regulatory ncRNAs is encoded in *cis,* which means that these ncRNAs are transcribed from the antisense strand of protein-coding genes [18]. Hence, they are complementary in full-length and can therefore form RNA duplexes with the mRNA of the targeted genes [19]. Most described examples of these *cis*-encoded antisense RNAs (asRNA) range from 100 to 300 nt in size, but some asRNAs are also shown to be substantially longer [18,20]. Antisense RNAs have been proven to either positively or negatively affect transcription, translation and mRNA stability [16]. In addition, a *cis*-encoded asRNA might work as a trans-encoded sRNA for another target [19]. Antisense transcription has been detected in multiple organisms [21] and, with the growing number of explored species, it is assumed that antisense transcripts can be found for ~10 to 20% of the bacterial genes [22]. A second class of RNA-based regulators encompasses *cis-*regulatory elements, mainly present at the 5′ untranslated region (5′UTR) of mRNA transcripts, e.g. riboswitches, T-boxes or thermosensors [23]. Whereas both 5′ as well as 3’untranslated regions can bear signals for the initiation and termination of translation [24,25], respectively, 5′UTRs additionally have the ability to fine-tune translation by *cis*-regulatory elements. They can be prone to RNA-binding proteins or antisense RNAs, carry attenuation systems [14,23] and play a role in mRNA stability [26]. In contrast, 3’UTRs are not as well understood and have escaped the attention of most transcriptomic studies [27]. It is known that long UTRs can be localized antisense to adjacent genes on the opposite strand; in fact some of these overlapping UTRs have been demonstrated to act as negative regulators for genes encoded on the opposite strand [20].

The development of next-generation sequencing techniques including RNA sequencing (RNA-Seq) enabled the genome-wide identification of RNA-based regulatory elements in an unprecedented depth. The high dynamic range of RNA-Seq allows the identification of transcripts which are expressed at vastly different levels. Also, this method does not exhibit background noise and is therefore appropriate for the identification of lowly abundant transcripts [28]. RNA-Seq analyses targeting ncRNA in particular, have been published for e.g. *Mycobacterium tuberculosis*[29], *Streptomyces coelicolor*[30] and *Sinorhizobium meliloti*[31].

The major goal of the project in which this study is embedded is the improvement of production strains and thus ultimately the enhancement of enzyme production. This study is targeted on the identification of active regulatory RNA elements within the different phases of a productive fermentation process. Therefore samples from crucial stages of an industrial-oriented *B. licheniformis* subtilisin fermentation process have been examined by strand-specific RNA-Seq and differential RNA-Seq (dRNA-Seq) [32]. A comprehensive analysis of the data revealed a multitude of RNA features which correlate to the physiology and the growth phases during the process. The combination of genomic data and RNA features provides an excellent basis to understand the regulatory events within an industrial fermentation process.

## Results and discussion

**Figure 1:**
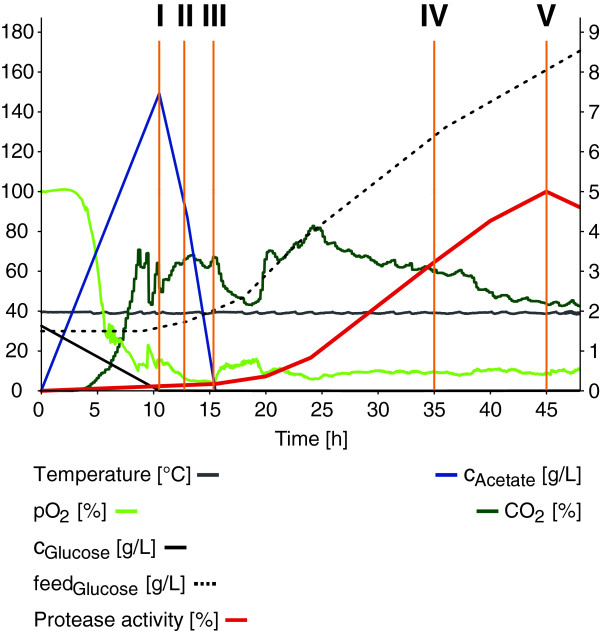
**Protease production and process parameters.** Process parameters are shown for fermentation L (the parameters for the replicate fermentations R and M are corresponding, data not shown). Temperature T [°C], oxygen partial pressure pO_2_ [%], glucose concentration c_Glucose_ [g/L], supplied glucose feed_Glucose_ [g/L] and normalized protease activity [%] are displayed on left y-axis, whereas acetate concentration c_Acetate_ [g/L] and carbon dioxide content CO_2_ [%] are scaled on the right y-axis. Process time t [h] is given on the x-axis. The sampling points I to V are indicated by orange lines.

*B. licheniformis* MW3Δspo, a germination deficient mutant of *B. licheniformis* DSM13, transformed with an expression plasmid encoding an alkaline serine protease, was grown in fed-batch mode in 6 L cultures. The fermentations were carried out in complex amino acid broth under conditions resembling the parameters used in industrial fermentation processes (Figure 1). To enhance the reliability of the analysis, the experiments were carried out in triplicate (L, R and M). Samples were taken at five selected time points of the fermentation process, which were chosen to follow the initial cell growth (sampling points I, II and III) and to determine the decisive changes within the early (IV) and the late stage (V) of the protease-producing states (Figure 1). Total RNA from each sample was prepared for strand-specific whole transcriptome sequencing [33]. RNA from samples L-I to L-V was additionally prepared for differential RNA-Seq for determination of transcription start sites (TSS), as described by Sharma et al. [32].

### Whole transcriptome sequencing

Strand-specific deep sequencing of the whole transcriptome of 15 *B. licheniformis* samples yielded more than 500 million reads with a specific length of 50 nucleotides. The number of reads for each library ranged from 2.4 × 10^7^ to 4.3 × 10^7^.

After the application of a strict quality processing (see Methods), 77.3 to 93.9% of these reads have been found to map to the chromosome and the expression plasmid used in this study (for details see Additional file 1: Figure S1 and Additional file 2: Table S1). Due to repeat regions, 1.45% of the *B. licheniformis* genome is not precisely mappable when considering the applied read length of 50 nucleotides. Thus, all reads mapping completely to such repeat regions have been excluded from further analysis. This pertains mainly to those 68.5 to 88.8% of reads which map to tRNA and rRNA genes. The majority of these rRNA matching reads can be assigned to 5S rRNA genes, which is in accordance with the fact that the applied depletion targets especially 16S and 23S rRNAs. Also, all reads mapping to the plasmid were removed from the dataset, as this analysis is focused on the transcriptional activity of the chromosome. Finally, 4.4 to 12.0% of the initial reads were taken for further analyses. These reads enabled the identification of transcriptional units and the determination of their boundaries to assign the transcriptional activity of coding as well as non-coding regions of the chromosome (see Methods).

To facilitate the comparison of different transcription levels between samples, we introduce the **n**ucleotide activity **p**er **k**ilobase of exon model per **m**illion mapped reads (NPKM) value as single nucleotide-resolution measure of transcriptional activity (see Methods). NPKMs for each RNA feature and for every gene were calculated and are available at Additional file 2: Table S2 and Table S3.

### Transcription start site determination and operon prediction

Differential RNA-Seq (dRNA-Seq) has been designed by Sharma et al. [32] to allow selective enrichment of native 5′ ends of transcripts for the determination of transcription start sites (TSS). The method is based on the observation that 5′ triphosphorylated RNA fragments are originating from native 5′ ends. In contrast, 5′ monophosphorylated RNAs are products of RNA decay or processing and do not contain information of transcription initiation. The dRNA-Seq approach includes a treatment with 5′ phosphate-dependent exonuclease (TEX), which results in the depletion of all monophosphorylated transcripts. It has been shown that TSS identification based on dRNA-Seq data is superior to an estimation of transcript boundaries based on whole transcriptome RNA-Seq reads [32].

**Figure 2:**
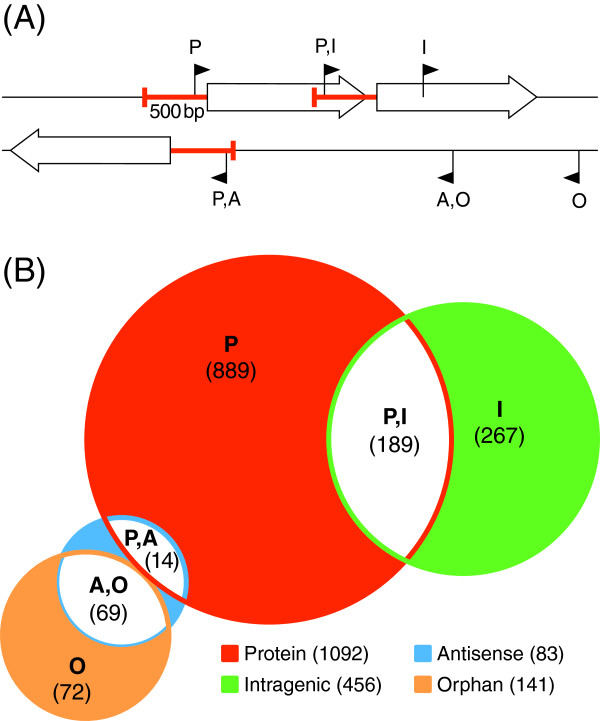
**Classification and distribution of TSS. (A)** Classification scheme of transcription start sites adapted from Dötsch et al. [34]. White arrows indicate genes. P: Protein-coding gene-dependent TSS located within a 500 bp range upstream of annotated start codons. I: Intragenic TSS situated within an annotated gene on the same strand. A: TSS localized antisense to an annotated gene. O: Orphan TSS not located in a promoter region or a gene on the same strand. **(B)** Distribution of transcription start sites identified in this study. Numbers in brackets give the amount of instances for each class. Numbers in the legend give the total amount of every class.

The differential sequencing of samples L-I to L-V resulted in 22,047,373 reads (Additional file 2: Table S4). A total of 2522 putative TSS was predicted (see Methods), 1500 of which were detected in at least two samples (Additional file 2: Table S5). A comparison of the latter with the transcript boundaries obtained by whole transcriptome sequencing (Additional file 2: Table S6) shows that 412 identified TSS confirm the RNA-Seq data, whereas the other findings introduce TSS not detectable by conventional RNA-Seq. To allow the assignment of the identified TSS to their putative origin, an allocation to four different classes was accomplished (Figure 2A) [34]. Naturally, the affiliation of TSS according to this schema is ambiguous as some TSS sort to multiple classes, e. g. some TSS are located in a promoter region and within the upstream gene as well. The distribution of the identified TSS to each class is shown in Figure 2B. 1092 TSS were detected in promoter regions, 72 genes are bearing more than one putative TSS in this region. The dRNA-Seq data enabled conclusions for TSS determination in cases in which read-through transcription of the upstream gene caused by leaky termination prohibits the identification of downstream TSS by conventional RNA-Seq data. Of the identified 456 intragenic TSS, 267 are not located in the 500 bp promoter region of the downstream gene, reflecting a high number of putative internal promoters. Orphan TSS may indicate potential start sites of yet unknown genes or non-coding RNAs, this is supported by the finding that 76 of the 141 detected orphan TSS could be allocated to identified ncRNAs.

Operon prediction based on RNA-Seq and dRNA-Seq data resulted in 2510 putative operons structuring the genome of *B. licheniformis* (Additional file 2: Table S7). While most operons are monocistronic (66.8%) or bicistronic (18.3%), seven operons seem to encompass more than ten genes (Additional file 1: Figure S2). This small number of long operons is not in accordance with the operon prediction made by Kristoffersen et al. [35] for *B. cereus*. The difference is due to the varying operon concept employed here. Especially the consideration of internal TSS in combination with distinct shifts of expression resulted in an increase of shortened operons in this study.

### Reannotation

**Figure 3:**
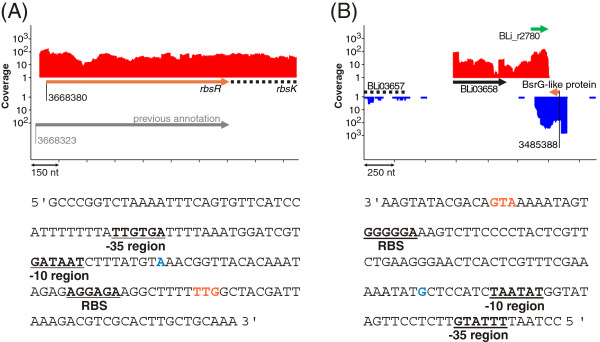
**Correction and insertion of annotated genes. (A)** Correction of start codons. *(Upper panel)* Transcriptional activity of *pooled RNA-Seq data*. The grey arrow displays the coordinates of the ribose operon repressor RbsR (BLi03840) according to Veith et al. [1]. Based on the transcriptional data, the start codon has been reassigned 57 bp downstream of the former position (orange arrow). *(Lower panel)* The new start codon is marked in orange and the transcription start site in blue. The location of patterns of a ribosomal binding site and -10 and -35 regions of the *rbsR*- regulating σ^A^ upstream of the gene provide additional confirmation to the new annotation. **(B)** Insertion of new genes. *(Upper panel)* Transcriptional activities (sample L-I) of BLi03658 (black arrow) and *indep* RNA BLi_r2780 (green arrow). The previously not detected [1,9] protein gene BLi05038 (orange arrow) was annotated as BsrG-like peptide (see also chapter *Comparative transcriptomics*). *(Lower panel)* The start codon of the new gene is marked in orange, and the transcription start site in green. The location of patterns of a ribosomal binding site and a σ^A^ -10 and -35 promoter region provide additional confirmation of the new annotation.

The first genome annotation of *B. licheniformis* DSM13 has been published in 2004 [1]. It has been shown previously that mapping of RNA-Seq data to genomes allows the correction of open reading frames and supports the identification of not-annotated protein genes [36]. Therefore, we performed a complete reannotation of the genome in order to integrate the RNA-Seq data provided by this study as well as the progress in gene prediction and annotation of the recent years. Distinct transcription start sites determined by dRNA-Seq and RNA-Seq-based whole transcriptome data have been used to identify putative mis-annotated genes (Figure 3A). These findings were validated by length comparisons to genes deposited in public databases and confirmation of ribosomal binding sites and -10 and -35 promoter regions. This approach enabled the correction of reading frames of 23 protein genes, 25 pseudo genes, 21 rRNA genes and two tRNA genes (Additional file 2: Table S8). Moreover, 60 previously not-annotated protein genes were identified based on transcriptional activity and protein conservation (Figure 3B, Additional file 2: Table S9). 52 genes (Additional file 2: Table S10) were removed from the annotation as these previously predicted ORFs could not be verified by detailed genome analysis and comparisons to public databases. In total, the reannotation approach resulted in a dataset containing 4297 ORFs. Comparisons to the annotation of *B. licheniformis* DSM13 by Rey et al. [9] showed that 16 of the newly annotated genes have not been described for this organism before. 18 of the removed genes were annotated in both genomes. More than 2000 gene annotations have been improved. These improvements mainly comprise former hypothetical proteins now assigned to a function and proteins with altered gene symbols. In addition to gene-associated improvements, seven genomic regions were identified as prophage regions based on GC content deviations, significant similarities to known prophage genes and the presence of insertion repeats. The transcriptional activity of the prophage regions was rather low, which is consistent with the observation that many prophages are induced during SOS response, which should not occur within a fermentation process [37].

### 5′ and 3′untranslated regions

**Figure 4:**
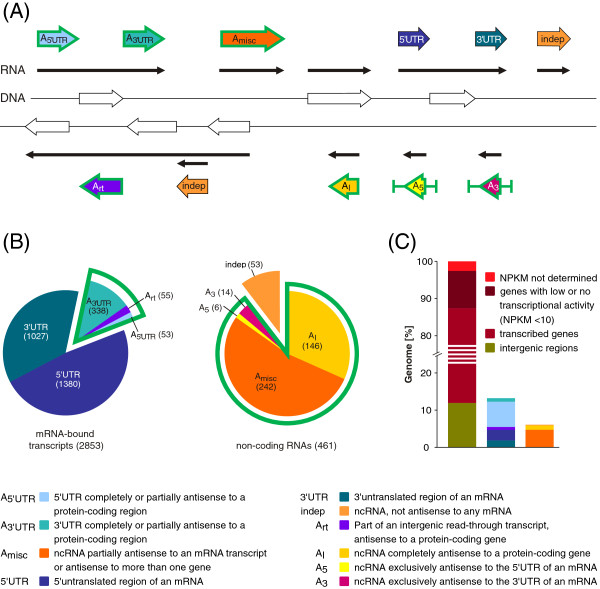
**Classification and distribution of RNA features. (A)** Classification scheme of ten RNA feature classes. White arrows indicate CDS and black arrows represent RNA transcripts. All antisense transcripts are framed green. RNA features which are part of an mRNA are denoted *5’UTRs* or *3’UTRs*. Antisense transcripts that are mRNA-bound were classified as *A*_*5’UTR*_, *A*_*3’UTR*_ and *A*_*rt*_*.* Non-coding antisense transcripts were classified as *A*_*5*_, *A*_*3*_ and *A*_*I*_ and comprise antisense transcripts opposite to 5’ and 3’UTRs or to protein-coding regions of the mRNA. *A*_*misc*_ designates antisense ncRNAs that target more than one gene or are only partially antisense. Independently transcribed ncRNAs without any antisense localization are designated *indep*. **(B)** Quantitative affiliation of identified RNA features. **(C)***(Left)* Proportional distribution of intergenic regions or annotated genes with different transcriptional activities within the complete *B. licheniformis* DSM13 genome. *(Middle + Right)* Percentage of the total genome covered by the defined RNA classes.

**Figure 5:**
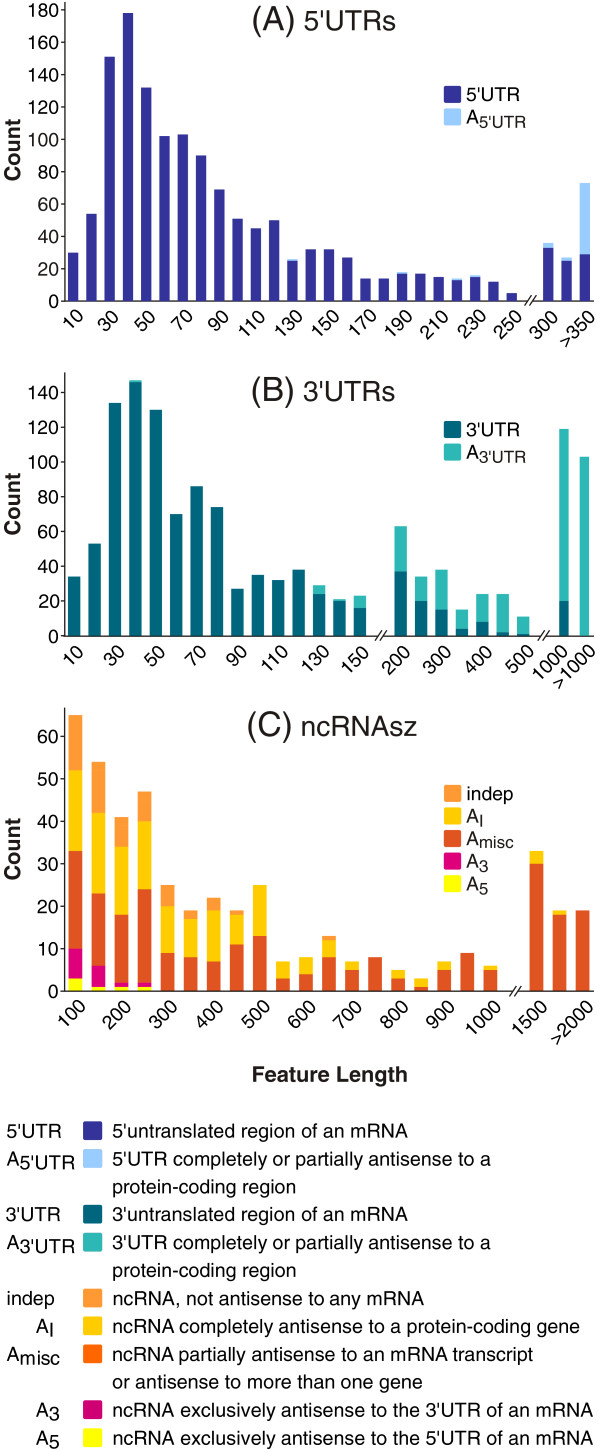
**Length distribution of RNA features.** Size range of **(A)** 1433 identified 5’ untranslated regions, **(B)** 1365 identified 3’ untranslated regions and **(C)** 461 identified non-coding RNAs. Please note that the classification scheme corresponds to Figure 4.

In this study, 1433 5′untranslated regions (Figure 4) with a mean length of 117 nt (Figure 5A, Additional file 2: Table S6) could be identified. Thirty of these 5′UTRs are shorter than 11 nt, implicating that leaderless transcription, commonly found in many bacteria [38], is not an abundant mechanism in *B. licheniformis.* Correspondingly, low occurrence of leaderless transcription has also been suggested for other members of the phylum *Firmicutes*[39]. The most strongly transcribed 5’UTRs ≥150 nt and the 5’UTRs discussed in the following passage are listed in Table 1.

**Table 1:** Selected 5’untranslated regions (5'UTRs)

**RNA feature**	**Start**	**Stop**	**Length**	**Downstream gene**	**Antisense genes**	***cis*-regulatory element**	NPKM value*
BLi_r0085	210580	210926	347	*thrZ*		T-box	1966
BLi_r0356	542296	542520	225	BLi00536		*ydaO-yuaA* leader	1368
BLi_r0498	712264	712587	324	*yybP*		*yybP-ykoY* leader	1144
BLi_r0691	942026	942290	306	*thiC*		TPP	5030
BLi_r0744	998913	999033	121	*glpD*			5693
BLi_r0943	1207762	1207542	180	*yitJ*		SAM	2782
BLi_r0982	1243825	1243498	328	*trpS*		T-box	865
BLi_r0983	1244045	1244232	188	*oppA*			1465
BLi_r1011	1271178	1271391	255	*tenA*		TPP	9963
BLi_r1028	1291984	1292289	306	*metI*		SAM	3605
BLi_r1168	1487545	1487279	226	*mtnK*		SAM	2207
BLi_r1196	1510226	1511237	1012	BLi05023	BLi01539, BLi01540		651
BLi_r1485	1973018	1973209	192	BLi02027			1035
BLi_r1609	2118173	2117083	1091	*gltA*	*gltC*		34
BLi_r1634	2146236	2145933	304	*expZ*			1225
BLi_r1709	2204969	2205111	143	*dhaS*			2816
BLi_r1801	2295779	2295571	168	*xpt*		Purine	1540
BLi_r1835	2356768	2356478	291	*hbs*			3478
BLi_r1850	2382265	2381914	352	*ribU*		FMN	2367
BLi_r1871	2409288	2409010	238	*ribD*		FMN	2512
BLi_r2045	2616129	2615830	300	*glyQ*		T-box	787
BLi_r2142	2742406	2742106	301	*yrzI*			860
BLi_r2241	2878256	2877902	313	*lysC*		Lysine	751
BLi_r2286	2949768	2949565	204	*citZ*			1563
BLi_r2389	3060789	3060456	292	*leuS*		T-box	998
BLi_r2510	3188213	3185988	2226	*kapD*	*yuxJ, pbpD*		13
BLi_r2628	3302655	3302393	221	*metN2*		SAM	2661
BLi_r3184	4014316	4014539	224	*yxjG*		SAM	5296
BLi_r3195	4037045	4036819	185	BLi04205		TPP	9303
BLi_r3196	4037110	4037236	127	BLi04206			2693

*(pooled RNA-Seq data).

**Figure 6:**
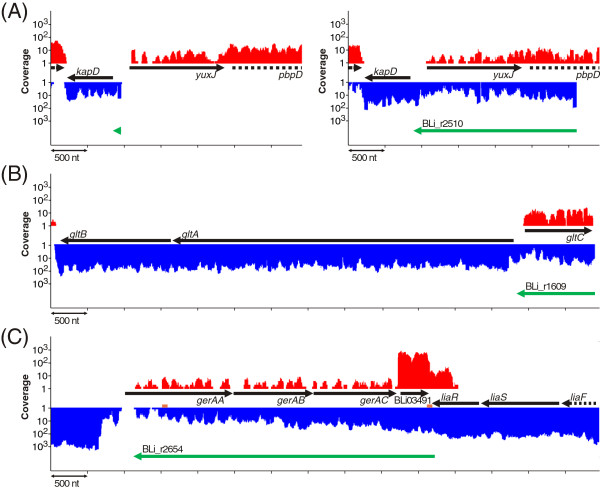
**Untranslated regions (UTRs).** Transcriptional activities of UTR regions. Black arrows indicate genes and green arrows the identified UTRs. **(A)** 5’UTR of *kapD* at sampling point II (left) and sampling point IV (right). **(B)** 5’UTR BLi_t1609 at sampling point IV. **(C)** 3’UTR BLi_r2654 (*pooled RNA-Seq data*). Predicted terminator sequences are marked orange.

At sampling points I, II, and III, the gene of the sporulation inhibitor KapD (BLi03329) reveals a 5’UTR of 113 nt (Figure 6A), whereas an alternative, dRNA-Seq-supported 5’UTR (BLi_r2510) with a length of 2226 nt is present at the later stages of the fermentation process. The TSS of both 5’UTRs seem to be preceded by a σ^A^ recognition site. At sampling point IV, the transcriptional activity of the gene is higher than the activity of the 5’UTR region. In *B. subtilis*, growth phase-dependent differentiation into subpopulations of distinct cell types with different gene expression patterns is well described [40,41]. The divergent expression levels of *kapD* and the long 5’UTR in *B. licheniformis* might therefore result from different usage of promoter sites dependent on the respective cell type. However, the observed effect could also derive from slow decay rates of the short form of the *kapD* mRNA transcribed earlier. 52 further 5’UTRs exhibited antisense activity towards upstream genes (*A*_*5’UTR*_; Figure 4), as shown for the untranslated region BLi_r1609 upstream of the glutamate synthase operon *gltAB* (BLi02161/62; Figure 6B). The observed 5’UTR is completely antisense to the gene of the corresponding transcriptional activator GltC (BLi02163). The dRNA-Seq data suggest the presence of only one TSS. This finding might be an example for a regulatory linkage between adjacent genes localized on different strands. This concept has recently been termed the excludon by Sesto et al. [20], who demonstrated that long 5’UTRs can act negatively on the transcription of the opposite gene. Following this idea in the case of the glutamate synthase operon, the preceding 5’UTR might establish a negative feedback regulation of the transcriptional activator GltC. A corresponding elongated UTR of the *gltAB* operon has not been found in *B. subtilis*[42,43], which indicates different regulations of glutamate homeostasis in the two species.

Next to regulatory effects based on antisense orientation, 5’UTRs can bear intrinsic, so-called *cis*-regulatory elements [44]. At the time of this study, 62 *cis*-regulatory elements have been predicted for *B. licheniformis* DSM13 by covariance models [45,46]. All elements have been shown to be transcriptionally active during the fermentation process (Additional file 2: Table S11), although some are not located in 5’UTRs but in intergenic read-through regions. Three new T-boxes, located upstream of the serine acetyltransferase gene *cysE* and the tRNA ligase subunit genes *glyQ* and *pheS,* could be identified by comparison to the Rfam database. In *B. subtilis*, 92 *cis*-regulatory elements have been described [43], comprising RNA switches as well as protein-binding RNAs. For 76 of these instances, transcription could be shown at orthologous loci in *B. licheniformis* (Additional file 2: Table S12).

1365 3’UTRs (Additional file 2: Table S6) with an average length of 276 nt have been identified according to Figure 4, the most strongly transcribed 3’UTRs ≥150 nt and the 3’UTR discussed in this chapter are listed in Table 2. Of the identified 3’UTRs, 42% exceed 100 nt and 16% even exceed 500 nt in length (Figure 5B). In total, 338 3’UTRs are localized antisense to adjacent genes (*A*_*3’UTR*_; Figure 4). A detailed manual inspection revealed that all 3’untranslated regions longer than 1000 nt seem to be protruding after incomplete termination [18,32]. Altogether, 684 3’UTRs with internal termination sites could be determined, whereas 511 3’UTRs end at predicted termination sites. These findings suggest that the effect of fading-out at the end of operons due to imperfect termination might be a common effect in *B. licheniformis*. An example is the 3965 nt 3’UTR (BLi_r2654) downstream of the cell envelope stress response operon *liaIHGFSR* (BLi03492-97; Figure 6C). The mRNA transcript of this operon protrudes beyond a termination signal, which is located directly behind the stop codon of *liaR*. This protruding mRNA sequence is antisense to the next four genes which comprise the germination receptor operon *gerAAABAC* (BLi03488-90) and a hypothetical protein (BLi03491). A second terminator structure can be found 370 nt upstream of the end of the transcript.

**Table 2:** Selected 3’untranslated regions (3'UTRs)

**RNA feature**	**Start**	**Stop**	**Length**	**Upstream gene**	**Antisense genes**	NPKM value*
BLi_r0075	198675	198433	243	*citM*		127
BLi_r0671	919040	918595	446	*ygzB*	*perR1*	50
BLi_r0688	938151	938591	441	BLi00936		496
BLi_r0817	1054694	1054523	172	*msmX*	BLi01051	75
BLi_r0859	1099760	1099445	316	*ynzH*	*yhfE*	2310
BLi_r0949	1209837	1210136	300	BLi01196		188
BLi_r1013	1278149	1277370	780	*cotZ*	*fabI, cotO*	116
BLi_r1145	1465905	1468238	2334	*ykoM*	*ykoU, ykoV*	264
BLi_r1333	1655028	1654895	134	*ylaL*		195
BLi_r1357	1680492	1679988	505	*ylbP*	*gerR*	62
BLi_r1521	2008172	2008031	142	BLi02067		224
BLi_r1720	2216049	2215342	708	*odhB*	*yocS*	41
BLi_r1750	2244661	2244519	143	*yodL*	*yoyE*	16
BLi_r1797	2291386	2291184	203	*ypbQ*		268
BLi_r1927	2463046	2465026	1981	BLi02544	BLi02545,*ymaC*	213
BLi_r1985	2537707	2537586	122	*mntR*		151
BLi_r1995	2550986	2550862	125	*tasA*		482
BLi_r2041	2606579	2606461	119	*cccA*		431
BLi_r2067	2662102	2661914	189	BLi02768	*yrhD*	87
BLi_r2141	2741955	2741415	541	*yrzI*		348
BLi_r2152	2754987	2754859	129	*yrzB*		140
BLi_r2178	2787395	2787201	195	*yrbF*		1520
BLi_r2292	2952407	2952263	145	*pyk*		520
BLi_r2582	3255915	3256420	506	*yutI*	*yuxL*	324
BLi_r2620	3292613	3292481	133	*sufB*		405
BLi_r2654	3332662	3328698	3965	*liaR*	*gerAA, gerAB, gerAC,* BLi03491	11
BLi_r2700	3398622	3398373	250	*copA*		166
BLi_r2729	3427607	3428159	553	BLi05033		345
BLi_r2752	3464447	3464713	267	BLi03635		286
BLi_r2855	3591778	3591637	142	*cccB*		80

*(pooled RNA-Seq data).

### Non-coding RNA features

Non-coding RNAs were identified in non-coding regions of the chromosome, for example in intergenic regions or localized in antisense direction to protein genes (see Methods). The boundaries of the identified transcripts were determined by upshifts or downshifts of transcriptional activity. All identified RNA features were checked for similarities to complete protein genes as well as protein domains to ensure that they indeed represent non-coding RNAs.

**Figure 7:**
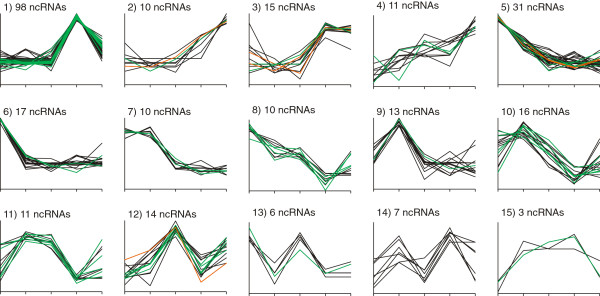
**Cluster analysis of ncRNA expression profiles.** Expression profiles of ncRNAs after *k*-means clustering (Additional file 2: Table S13). The x-axis shows sampling points I to V from left to right and the y-axis gives the expression strength in z-score transformed mean NPKM values of each replicate. Clusters are numbered and captioned with the count of included ncRNAs. Transcripts with a maximal NPKM value >100,000 are marked in orange and transcripts with a maximal NPKM value >1000 are marked in green.

To extract the basic types of ncRNA expression profiles during the examined fermentation process, cluster analysis based on the *k*-means algorithm [47] was applied to those 273 ncRNAs with highly reliable replicates. In total, 15 clusters of divergent expression profiles were generated (Figure 7, Additional file 2: Table S13). Cluster 1 contains 36% of the applied ncRNAs and 50% of all ncRNAs >1000 nt. It displays a strong up-shift of transcriptional activity at sampling point IV followed by a decrease at sampling point V. The high portion of transcripts in this cluster prompts the conclusion that RNA-based regulation is especially important during the later stages of the fermentation process. Other ncRNAs exhibiting up-shifts of transcriptional activity are displayed in clusters 2 to 4, whereas clusters 5 to 8 include transcripts with activity down-shifts. The further clusters comprise ncRNAs with expression shifts during the early fermentation process, as well as an activity up-shift at sampling point V in clusters 10 to 12.

All assigned non-coding RNAs were categorized according to the scheme displayed in Figure 4 and subdivided into the classes *A*_*5*_, *A*_*3*_, *A*_*I*_, *A*_*misc*_ and *indep*. Selected ncRNAs are listed in Table 3, whereas an overview of all identified features is given in Additional file 2: Table S6. Several ncRNAs have been selected for validation by Northern blotting (Additional file 1: Figure S3). The analyzed ncRNAs were chosen as they are exemplarily for their respective class. The occurrence of eight ncRNAs could be verified, especially ncRNAs <500 nt are in good accordance with the results gained by RNA-Seq. The results for transcripts >2000 nt are indicative for RNA degradation or processing and leaky transcription termination. However, three ncRNAs could not be validated, which is most probably due to their low expression levels.

**Table 3:** Selected non-coding RNAs (ncRNAs)

**RNA feature**	**Start**	**Stop**	**Length**	**Class**	**Rfam**	**Upstream gene**	**Downstream gene**	**Antisense genes**	NPKM value*
BLi_r0016	30440	30837	398	*indep*	Scr	*tadA*	*dnaX*		223117
BLi_r0026	54282	49490	4793	*A*_*misc*_	RnaA			*metS, yabD, yabE, rnmV*	18
BLi_r0086	212998	213153	156	*indep*		*thrZ*	BLi00235		242952
BLi_r0253	413569	412086	1484	*A*_*misc*_				BLi00412, BLi00413	14
BLi_r0415	617488	617027	462	*A*_*misc*_				*thiL*	6
BLi_r0451	653178	652888	291	*A*_*misc*_				BLi00649	15139
BLi_r0844	1082413	1082091	323	*indep*		*yhaA1*	*hit*		8039
BLi_r0872	1108968	1109111	144	*A*_*I*_				*apr*	25689
BLi_r1000	1262387	1262504	118	*indep*	RsaE	*pepF*	*yjbL*		5791
BLi_r1034	1300088	1300311	224	*indep*		*pbpE1*	BLi01297		1902
BLi_r1306	1639742	1639946	205	*A*_*misc*_	SR1			*speA*	502
BLi_r1347	1673741	1673635	107	*A*_*misc*_	CsfG			*ylbG, ylbH*	5366
BLi_r1424	1898847	1898597	251	*indep*		*yqeD*	BLi01936		2023
BLi_r1454	1929530	1929709	180	*indep*	BsrB				26604
BLi_r1474	1960434	1960112	323	*indep*		BLi02008			61592
BLi_r1596	2101575	2102388	814	*A*_*I*_				*cysP2*	12
BLi_r1645	2156680	2156597	84	*indep*		*yobS*	*yndG*		13545
BLi_r1808	2302203	2301803	401	*indep*	RnpB	*gpsB*	*ypsC*		55930
BLi_r1834	2355791	2356137	347	*A*_*misc*_				*folE*	1
BLi_r2049	2634028	2634231	204	*A*_*misc*_	SurC			*dnaK*	29
BLi_r2163	2770133	2769931	203	*indep*	BsrA	*aspS*	*yrvM*		389442
BLi_r2390	3060847	3062662	1816	*A*_*misc*_				*ytvB, yttB*	11
BLi_r2624	3299320	3299025	296	*indep*	BsrI	*yurZ*	BLi03452		514
BLi_r2645	3325524	3323636	1889	*A*_*3'UTR*_		*yirB*		*cssR, cssS*	112
BLi_r2758	3469610	3469009	602	*indep*	SsrA	*smpB*	BLi03638		78287
BLi_r2780	3485163	3485306	144	*indep*		BLi03658	BLi03670		3747
BLi_r2828	3552518	3552467	52	*indep*		*trxB*	*yvcI*		15924
BLi_r2863	3611192	3612078	887	*A*_*misc*_				*degU, degS*	5
BLi_r2925	3692599	3692663	65	*A*_*I*_				BLi03865	14110
BLi_r3203	4050815	4050900	86	*A*_*misc*_				*bglP*	35711

*(pooled RNA-Seq data).

#### *Indep* ncRNAs

**Figure 8:**
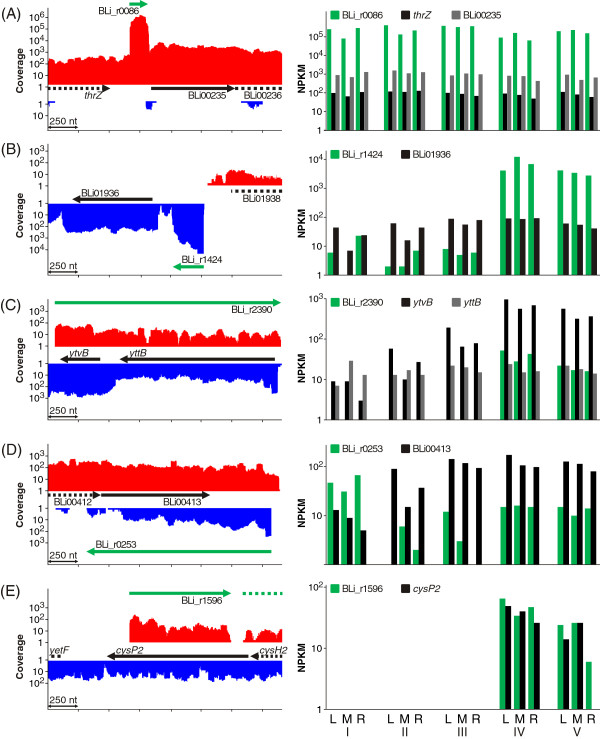
**Non-coding RNAs (ncRNAs).***(Left)* Sum of transcriptional activities from all 15 replicates (*pooled RNA-Seq data*). Black arrows indicate genes and green arrows the identified ncRNAs. *(Right)* Log-transformed NPKM values of ncRNAs and adjacent genes for single samples. **(A)***Indep* RNA BLi_r0086 is transcribed constitutively with a length of 156 nt and located between the genes of threonyl-tRNA synthetase (*thrZ*, BLi00234) and a hypothetical protein (BLi00235). Both adjacent genes are also transcribed constitutively, but are less abundant by four and three orders of magnitude, respectively. **(B)** The differentially expressed *indep* transcript BLi_r1424 is located between the gene of a hypothetical protein (BLi01936) and a pseudogene (*yobN*, BLi01938) with a length of 251 nt. The TSS could be confirmed by dRNA-Seq. In the three early conditions the BLi_r1424 transcription level is low, but NPKM values of more than 12,000 were recorded during the productive stages of the fermentation process. A direct transcriptional connection to the adjacent BLi01936 is not visible from the shown NPKM values. **(C)** BLi_r2390 antisense to *ytvB* (BLi03176) and *yttB* (BLi03177) is an example for long antisense ncRNAs. The *A*_*misc*_ RNA occurs only in the later stages of the fermentation process, parallel to a distinct increase in transcriptional activity of *ytvB*, but does not exceed it regarding the NPKM value. **(D)** One example suggesting a regulatory function of *A*_*misc*_ RNAs is BLi_r0253, oriented antisense to BLi00413. In the earliest stage the asRNA shows stronger transcription than the corresponding gene, but in all later stages the asRNA is only weakly transcribed. This might indicate a silencing effect in the exponential stage. **(E)***A*_*I*_ RNA BLi_r1596 localized antisense to the gene of the sulfate permease CysP2 (BLi02153). The transcription of both, the ncRNA and the protein-coding gene, starts during the late stages of the fermentation process.

As depicted in Figure 4, *indep* transcripts are defined as non-coding RNAs not localized antisense to any mRNA. Instead they can be found in intergenic regions or any other position of the chromosome. In total, 53 *indep* RNAs with sizes between 51 and 602 nt have been identified, of which 40 have TSS verified by dRNA-Seq. Within this group five housekeeping sRNAs could be annotated: the tmRNA SsrA (BLi_r2758), the 6S RNAs BsrA (BLi_r2163) and BsrB (BLi_r1454), the RNA component of RNase P RnpB (BLi_r1808) and the signal recognition particle Scr (BLi_r0016) [13]. 87% of the *indep* transcripts exhibited NPKM values ≥100 in at least three samples, reflecting a strong transcriptional activity of the encoding genomics regions. For example the sRNA Scr, an essential part of the protein secretion system [48], reaches a maximal NPKM value of almost 400,000. This is in perfect accordance with the fact that the cells are derived from a fermentation process optimized for protein secretion. Interestingly, 39 *indep* ncRNAs seem to be transcribed constitutively under the examined conditions (Figure 8A), whereas only thirteen *indep* RNAs show differential expression (likelihood value ≥0,99) [49]), as illustrated exemplarily for BLi_r1424 (Figure 8B).

#### Antisense ncRNAs

In contrast to the class of indep ncRNAs, the antisense ncRNAs (asRNAs) *A*_*I*_, *A*_*3*_, *A*_*5*_, and *A*_*misc*_ comprise non-coding transcripts localized antisense to annotated protein-coding genes. They either target the protein-coding region of a gene (*A*_*I*_) or the 5’ and 3’ untranslated regions (*A*_*5*_ and *A*_*3*_). Furthermore, ncRNAs that target more than one gene or that are only partially antisense are classified (*A*_*misc*_). In this study, 242 *A*_*misc*_ RNAs (Figure 8C/D) could be identified. Approximately 150 of them (and also all *A*_*5*_ ncRNAs) are located opposite to ribosome binding sites and could therefore function as inhibitors of translation, a very common mechanism of *cis*-encoded asRNAs [50]. The length distribution of the non-coding RNA features is shown in Figure 5C, and illustrates that 42% of the *A*_*misc*_ RNAs are less than 400 nt in length. Twenty-seven of these short *A*_*misc*_ RNAs reach maximal NPKM values ≥100, suggesting putative sRNA mechanisms. However, some *A*_*misc*_ RNAs are much longer, i.e. BLi_r2246 is 6348 nt in length and spans six genes. The occurrence of antisense transcripts of such length is not unexpected, as asRNAs with very diverse sizes, reaching more than 7000 nt, have been described for several species [18]. Furthermore, 146 *A*_*I*_ transcripts (Figure 8E) could be assigned, ranging in size from 54 to 1572 nt. Over 95% of the *A*_*I*_ transcripts exhibit maximal NPKM values ≤100, 68% even ≤20, due to the low coverage only 20 TSS could be verified by dRNA-Seq for these asRNAs. In total, 408 non-coding asRNAs were determined, comprising 89% of all identified non-coding RNA transcripts and targeting 15% of all genes. The number of identified antisense ncRNAs is in accordance to previous studies which assume that antisense transcription concerns ~10 to 20% of the bacterial genes [22].

### Antisense transcripts with putative impact on productivity

The general aim of our group is the identification of productivity related features. Thus, a special focus has been set on the identification of antisense transcripts with a putative impact on protease production as targets for strain improvement.

**Figure 9:**
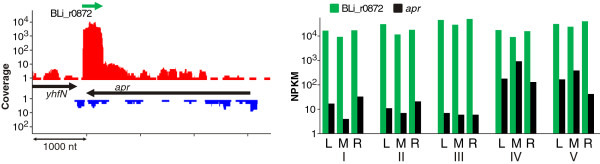
**Antisense RNA against Subtilisin Carlsberg.***(Left)* Transcriptional activities (sample L-III) of *apr*, the gene which encodes Subtilisin Carlsberg, and the *A*_*I*_ RNA BLi_r0872 (green), which is antisense to the 3’UTR of *apr*. *(Right)* Log-transformed NPKM values of BLi_r0872 and *apr*.

The alkaline serine protease Subtilisin Carlsberg (*apr,* BLi01109) represents the major secreted feeding protease of *B. licheniformis.* Thus, it competes for energetic and secretory resources with the production protease. We identified a *cis*-encoded 144 nt *A*_*I*_ asRNA (BLi_r0872) which is located at the 3’end of the *apr* mRNA (Figure 9). A highly active TSS determined from the dRNA-Seq data and a terminator structure downstream of the adjacent gene *yhfN* confirm the characterization of the transcript as independently transcribed asRNA. BLi_r0872 is highly expressed at all fermentation stages, whereas the transcriptional activity of the Subtilisin Carlsberg gene increases at the productive stages of the process. The presence of the *cis*-encoded asRNA opposite to the 3’end of the target mRNA resembles the *B. subtilis* RatA/*txpA* toxin/antitoxin system or the *Escherichia coli* GadY/*gadX* system in which an antisense RNA promotes either mRNA degradation or stability [19]. To elucidate the impact of the detected asRNA, further analyses will be necessary, especially as a corresponding transcript is absent in the transcriptome of *B. subtilis*[43].

Further antisense transcripts against genes involved in cell differentiation, cell stress response, and thiamine and folate biosynthesis could be observed and are presented in Additional file 1: Figure S4.

It is exciting to think about a regulatory impact of the mentioned ncRNAs, but there are also some noteworthy limitations to putative effects. (i) The completed RNA-Seq experiments cannot discern if the sense and the antisense transcript are transcribed in the same type of differentiated cells, which especially challenges stoichiometric estimations of asRNAs and their mRNA targets. Whether they can influence each other or fulfill different purposes in different cell types has to be a topic of single cell targeted investigations. (ii) It has been reported that functional sRNAs are produced in excess amounts over the targeted mRNA [16,51]. Therefore, a regulatory mechanism of poorly transcribed antisense RNA cannot be assumed *bona fide,* but has to be evaluated carefully. Nonetheless, our data implicate that there might be a biological function assignable to the RNA features, especially when they are conserved within related species as *B. subtilis*. (iii) At last, it has to be experimentally excluded, especially for low abundant instances, that the found ncRNAs originate from spurious transcriptional events, for instance driven by alternative sigma factors [43].

### Comparative transcriptomics

In total, we determined 461 candidate non-coding RNA transcripts, including antisense, as well as *indep* ncRNAs (see *Non-coding RNA features*). For *Synechocystis* sp. PCC6803, *Sinorhizobium meliloti* and the archaea *Sulfolobus solfataricus* P2 and *Methanosarcina mazei* Gö1 between 50 and 107 non-coding RNAs per Mb were identified [31,52-54], matching our result of 109 ncRNAs/Mb. For *B. subtilis*, the close relative of *B. licheniformis*, Nicolas et al. [43] have found 472 non-coding RNA features in a tiling array-based, condition-dependent transcriptome study. The majority (68%) of these features are intergenic transcripts determined by promoter analysis, whereas only 32% are derived from independently transcribed (antisense) RNAs. In contrast, the majority of ncRNAs identified in *B. licheniformis* are antisense RNAs (89%), transcribed independently from protein-coding genes. The identification of more antisense transcripts in *B. licheniformis* might be accounted to the reduced background noise in RNA-Seq in comparison to tiling arrays, which allows a better detection of low abundant transcripts [28]. 167 of the *B. licheniformis* ncRNAs are located in regions with high sequence similarity to *B. subtilis*[55] and 126 ncRNAs are encoded at the frontiers of conserved and not conserved regions of the two genomes. Based on sequence similarity, only 43 (Additional file 2: Table S14) out of the, in total, 293 ncRNAs located in these regions seem to occur in the *B. subtilis* transcriptome [43], emphasizing the differences of the two closely related species. Comparisons to two earlier *B. subtilis* transcriptome studies show similar low levels of accordance [56,57]. However, as mentioned above, it is also possible that the identified antisense ncRNAs partly derive from spurious transcription events [43], and hence do not introduce a species-specific effect.

For *B. subtilis*, 22 sRNAs have been validated experimentally [43,58]. Comparison to Rfam and/or comparison of genomic locations facilitated the detection of eleven of these sRNAs in the transcriptome of *B. licheniformis* (Additional file 2: Table S15). These include, in addition to the mentioned five housekeeping sRNAs [13], two regulatory RNAs with well-known function in *B. subtilis*: SR1 and RnaA [59,60]. The other RNAs found in *B. licheniformis* are BsrI, CsfG, SurC and RsaE [61-64]. The *B. subtilis* sRNAs which could not be confirmed in *B. licheniformis* originate from loci with no conserved gene pattern in this organism and thus may contribute to the differences between the two species. Jahn et al. [65] described the toxin-antitoxin system BsrG/SR4 located in the SPβ prophage region of *B. subtilis*. Although *B. licheniformis* does not harbor a homolog of the SPβ prophage, two distinct transcripts were found to encode peptides similar to the BsrG toxin (Additional file 1: Figure S5). Additionally, the transcriptional activity of the corresponding loci revealed pairs of overlapping transcripts from both strands (Figure 3 and Additional file 1: Figure S5) as shown for the BsrG/SR4 type toxin-antitoxin system. Therefore both newly identified ORFs were annotated as BsrG-like peptides (BLi05015 and BLi05038). Furthermore, the antisense transcripts (*indep* RNAs BLi_r1034 and BLi_r2780) resemble the SR4 antitoxin, especially in stem loops SL3, SL4 and TSL [65] directly antisense to the BsrG-encoding mRNA.

## Conclusions

**Figure 10:**
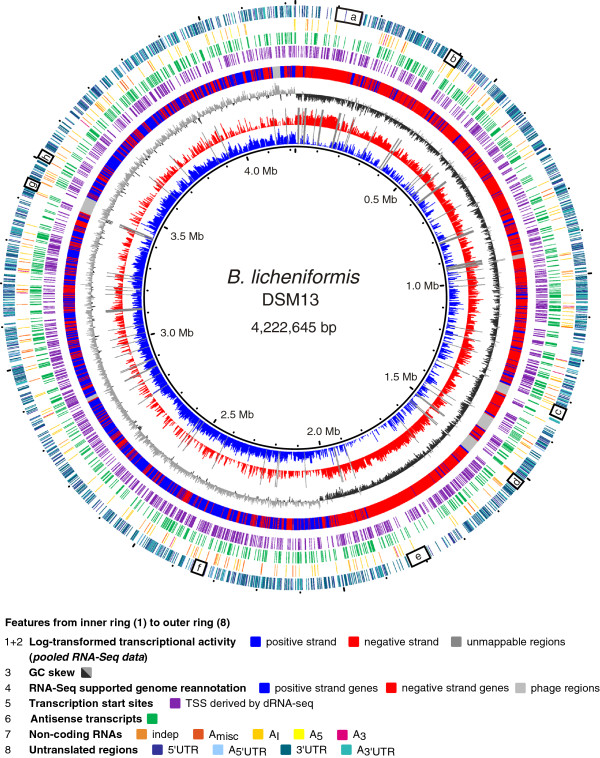
**Circular plot of transcriptional activity and identified RNA features.** Combined depiction of reannotated genes and transcriptional activity of *B. licheniformis*. Unmappable regions, GC skew, transcription start sites, non-coding RNAs, untranslated regions and antisense transcripts are also shown. 5’UTRs and 3’UTRs are evenly distributed over the whole chromosome of *B. licheniformis*, except for regions a – h: these regions contain long operon structures (a: ribosomal superoperon, b: *lch* operon, e: *fla/che* operon, f: *trp* operon, h: *eps* operon) or prophage regions with low transcriptional activity (c, d and g). The classification scheme corresponds to Figure 4. (*indep*) ncRNA, not antisense to any mRNA; (*A*_*misc*_) ncRNA partially antisense to an mRNA transcript or antisense to more than one gene; (*A*_*i*_) ncRNA completely antisense to a protein-coding gene; (*A*_*5*_) ncRNA exclusively antisense to the 5'UTR of an mRNA; (*A*_*3*_) ncRNA exclusively antisense to the 5'UTR of an mRNA; (5'UTR) 5'untranslated region of an mRNA; (*A*_*5'UTR*_) 5'UTRs completely or partially antisense to a protein-coding region; (3'UTR) 3'untranslated region of an mRNA transcript; (*A*_*3'UTR*_) 3'UTRs completely or partially antisense to a protein-coding region.

The presented study generated substantial data on the transcriptional activity of *B. licheniformis* within five relevant growth stages of an industrial-oriented fermentation process. A detailed analysis of the transcriptome data enabled us to accomplish a high quality functional genome reannotation of *B. licheniformis* DSM13 (Figure 10, Ring 4). The integration of the reannotation and the transcriptionally active regions (Figure 10, Ring 1&2) resulted in the identification and quantification of hundreds of RNA based regulatory elements as well as protein encoding genes.

In total, 3314 RNA features have been sorted into ten functional classes (Figure 4). 1433 5’UTRs and 1365 3’UTRs (Figure 10, Ring 8) as well as 461 ncRNAs (Figure 10, Ring 7) and 55 antisense intergenic read-through (*A*_*rt*_) transcripts have been identified. A striking observation was the identification of 855 RNA features, which mapped antisense to annotated genomic features (Figure 10, Ring 6). Notably antisense RNA features have been found in each of the functional classes and include transcripts of a size range from 38 to 6348 base pairs in length. We have identified both: constitutively as well as growth phase dependently expressed RNA features.

Our data represent a solid amount of knowledge on regulatory elements which orchestrate the cellular activities of *B. licheniformis* during the succession of growth phases within a productive fermentation. To generate an overview of the functional diversity of the identified RNA features, all instances have been screened against the Rfam database. This approach resulted in hits to experimentally well characterized RNA features known from *B. subtilis* and other relatives, as well as in a multitude of so far unknown RNA features without any Rfam hit. The knowledge on genes and regulatory RNA features which are transcriptionally active during an industrial-oriented fermentation enables an excellent access to a rational strain design approach for the optimization of *B. licheniformis* as industrial workhorse. Especially the regulatory features which represent differences to the model organism *B. subtilis* give new insights to the still open question what makes strains of the species *B. licheniformis* superior to *B. subtilis* strains in terms of protease production capacity in industrial applications [2]. In the future it may be promising to correlate the transcriptional activity of the RNA features to the corresponding protein expression patterns.

## Methods

### Bacterial strain and fermentation conditions

*Bacillus licheniformis* MW3Δspo (kindly provided by F. Meinhardt and St. Wemhoff, University of Münster) was used for the fermentation experiments. *B. licheniformis* MW3Δspo is a derivate of the *B. licheniformis* wild type strain DSM13, bearing three deletions: Δ*hsdR* and Δ*hsdR2* coding for restriction endonucleases [10] and Δ*yqfD*[66] to prevent the production of viable spores and thus the long-term contamination of the used fermenters [67].

Fermentation was carried out for 46 h in aerated 16 L fermenters with a culture volume of 6 L at 39°C. Medium contained 12% w/v of a complex nitrogen source, 57 mM KH_2_PO_4_, 21 mM (NH_4_)_2_SO_4_, 0.53 mM Mn(II)SO_4_, 0.17 mM Fe(II)SO_4_, 2.0 mM CaCl_2_ * 2 H_2_O, 5.7 mM MgSO_4_, 0.4% v/v PPG200, 0.03 mM tetracycline and 3% w/v glucose. The pH value was regulated to a set point of 7.9 with sodium hydroxide solution. Glucose-feed was started after exceeding the point of biphasic growth.

### RNA isolation and preparation

5 mL of the harvested cells were mixed with 5 mL of RNAprotect Bacteria Reagent (Qiagen) directly upon sampling. After 10 min incubation at room temperature the samples were centrifuged at 4500× *g*, the supernatant was removed, the sample was snap-frozen in liquid nitrogen and finally stored at -80°C. The cells were separated from the remainders of the fermentation broth by washing repeatedly with Buffer RLT (Qiagen). Subsequent RNA isolation was carried out with a modified protocol of the RNeasy Midi Kit (Purification of RNA including small RNAs using the RNeasy Midi Kit RY39 Apr-09, Qiagen) to retain short RNAs. The cells were disintegrated with the ball mill Mikro-Dismembrator U (B. Braun Biotech) in 400 μL Buffer RLT and afterwards resuspended in 1.4 mL Buffer RLT and 2.7 mL pure ethanol. The initial washing step of the column was done using 4 mL Buffer RWT (Qiagen). The DNA was digested successively with two different DNases (TURBO™ DNase, Ambion and DNase I recombinant, Roche), with a purification step after the first treatment. Purification was performed with a protocol adapted for small RNA purification of the RNeasy MinElute Cleanup Kit (Qiagen). Instead of 250 μL, 675 μL pure ethanol were added to the RNA before binding to the column to shift the binding capacity of the column. A control PCR with 35 cycles was conducted to confirm complete DNA removal. Depletion of rRNA was obtained using the MICROBExpress™ Bacterial mRNA Enrichment Kit (Ambion) according to manufacturer’s instructions. The following purification step was also carried out with the described adaption to the RNeasy MinElute Cleanup Kit.

### Library construction and sequencing

cDNA libraries were prepared by vertis Biotechnologie AG, Germany (http://www.vertis-biotech.com). For whole transcriptome libraries [28,33] the RNA samples were fragmented by ultrasound and dephosphorylated with Antarctic phosphatase. After polynucleotide kinase treatment the RNA was poly(A)tailed and an RNA adapter was ligated to the 5’phosphate. cDNA synthesis was accomplished by the use of poly(T) adapters and M-MLV reverse transcriptase. The subsequent PCR was carried out with cycle numbers between nine and twelve. The construction of the libraries for the dRNA-Seq was performed as described by Sharma et al. [32], supplemented by an additional treatment with polynucleotide kinase after the fragmentation step to allow removal of fragments previously not phosphorylated. The samples were incubated with Terminator™ 5’-Phosphate-Dependent Exonuclease (Epicentre) and a poly(A) tail was ligated to the 3’end of the transcripts. Hereafter an incubation step with tobacco acid pyrophosphatase and the ligation of an RNA adapter to the 5’end was conducted. Reverse transcription was processed as described above, the cycle numbers of the following PCR were 14 or 15. The RNA-Seq libraries as well as the libraries for dRNA-Seq were size fractioned in the range of 200 to 400 nt on agarose gels and then sequenced on an Illumina HiSeq 2000 machine with a read length of 50 nt.

### *In silico* sequence read processing

Initially, all sequence reads mapping to *B. licheniformis* rRNA and tRNA genes according to BLAST analysis were removed. The remaining reads were processed in a multi-step procedure to ensure the reliability of the read mappings used for the analysis of the transcriptional activity of the genome and to estimate the quality of the RNA-Seq data. All reads which mapped over the full read length of 50 bases with 98% or sequence identity were used for further analyses. Additionally, a distinct bit score was required to ensure an unambiguous assignment to one locus. All discarded reads were screened with relaxed similarity quality criteria vs. the *B. licheniformis* genome. 75% of these reads generated hits and were therefore assigned as bad quality *B. licheniformis* reads. The remaining reads (approximately 3% of the total generated sequence) cannot be mapped on the genome. A detailed sequence analysis of these unmappable reads revealed that they mainly contain poly(A) tails or concatenated adapters and therefore represent methodic artifacts. All datasets were depleted for plasmid-mapping reads and have been deposited in NCBI Sequence Read Archive database under accession number SRP018744 (Additional file 2: Table S16).

To obtain the maximal number of features, a dataset containing all reads of the 15 samples was prepared and is referred to as *pooled RNA-Seq data*. To reduce putative background noise, all reads with coverage of one and no intersecting or adjacent reads were omitted prior to combination of the datasets. This is done to reduce transcriptional activity that was not replicated within a dataset in order to avoid incorrect extension of predicted features (e.g. transcriptional activity from leaky termination masking or extending 5’UTR extensions due to overlap). The generation of five datasets describing each sampling point was processed accordingly.

#### Expression strength values

The analytical methods used to process the 15 generated RNA-Seq datasets require the use of single nucleotide activities instead of read mappings. This makes RPKMs [68] inapplicable as a measure of transcriptional activity. Instead, we defined the **n**ucleotide activity **p**er **k**ilobase of exon model per **m**illion mapped reads (NPKM) value. An NPKM is defined as:

$$\mathrm{NPKM}\;\left(n,m\right)=10^{9}\frac{\sum_{i=n}^{m}f\left(i\right)}{\sum_{i=1}^{m}g\left(i\right)\left(m-n\right)}$$

Where *n* and *m* are the start and stop of the region of interest, *f(i)* is the base activity of base *i* on a specific strand and *g(i)* is the sum of the activities of base *i* of positive and negative strands.

NPKM values are a derivate of RPKMs [68], adapted to per base nucleotide activities. They are designed to be functionally equivalent to RPKMs, albeit they are more accurate due to the single base-resolution. We are aware that RPKMs and therefore NPKMs do not account for sequencing-based bias [69]. Although sequencing-based bias produces some local errors, the overall comparability of active genomic regions is still possible.

#### Untranslated regions

5’ and 3’ UTRs were considered as regions of continuous, non-interrupted transcriptional activity upstream or downstream of annotated genomic features, respectively. The boundary of an identified 5’UTRs was set at the point of the rising of the continuous transcript from zero transcriptional activity. The boundary of a 3’ UTRs was accordingly set at the point of the downshift of the continuous transcript to zero transcriptional activity.

The analysis of 5’ and 3’untranslated regions was aimed to find the longest UTR, as the longest transcript should cover all possible alternative UTRs and contain all transcribed regulatory elements. Therefore, the computational analysis was based on the *pooled RNA-Seq data*. Few 5’ and 3’UTRs were manually extended on account of adjacent transcripts which are only separated from the UTR by a very short downshift and potentially are part of the UTR. To exclude that the resulting UTRs correspond to previously not annotated protein genes, searches versus the InterPro and the UniProtKB/Swiss-Prot databases were performed [70,71].

5’ and 3’UTRs which are antisense to an adjacent gene on the opposite strand were classified as *A*_*5’UTR*_ and *A*_*3’UTR*_. The respective UTRs were computationally examined and assigned to be antisense when their overlap to an opposite gene exceeded 100 nt in length.

Intergenic read-through transcripts localized antisense to an opposite gene were determined manually and classified as *A*_*rt*_.

#### Non-coding RNA features

The RNA-Seq data were scanned for transcriptionally active regions that were clearly separated from the transcripts corresponding to any annotated gene or its untranslated regions. This primary computational search identified transcripts which were either located on the opposite strand of a protein-coding gene, in intergenic regions or any other region of the chromosome. The boundaries of the identified transcripts were set to those nucleotides with the first and last occurrence of transcriptional activity higher than zero of the corresponding transcriptional unit. NPKM values for the resulting loci were generated from each of the 15 datasets. Subsequently, all results from the computational search were evaluated as depicted in Additional file 1: Figure S6A to approve the reliability of the identified ncRNAs. Searches vs. the InterPro and the UniProtKB/Swiss-Prot databases were performed to exclude the possibility that the resulting non-coding RNA features correspond to non-annotated protein genes [70,71]. Subsequently, the non-coding transcripts were subdivided into the ncRNA classes described in Figure 4 (*A*_*3*_, *A*_*5*_, *A*_*I*_, *A*_*misc*_, and *indep*).

The class *indep* comprises all identified ncRNAs that are not located antisense to any protein-coding gene or its respective untranslated regions. Several transcripts of this category were added manually as this class comprises RNA transcripts which could not clearly be distinguished from surrounding mRNAs by complete down-shifts of transcriptional activity, but were detected by their remarkably higher abundance.

The categories *A*_*3*_, *A*_*5*_, *A*_*I*_ and *A*_*misc*_ comprise ncRNAs which are localized antisense to protein-coding genes or their respective untranslated regions. The class *A*_*I*_ contains all ncRNAs with an antisense localization solely towards a protein-coding gene. The class *A*_*5*_ contains all ncRNAs with an antisense localization solely towards the 5’UTR of an opposite mRNA. The class *A*_*3*_ contains all ncRNAs with an antisense localization solely towards the 3’UTR of an opposite mRNA. The class *A*_*misc*_ contains all ncRNAs with an antisense localization towards more than one protein-coding gene and all ncRNAs which are only partially antisense to an mRNA transcript.

#### Analysis of dRNA-Seq reads

Transcriptional start sites were determined by the identification of significant increases of the log-scaled expression strength of the dRNA-Seq data from succeeding bases greater than ln 4. The reference value of ln 4 was empirically determined based on the observation that ln 4 represents the smallest expression strength increase for TSS present across all samples of one sampling point. In a second step, all TSS in promoter regions of rRNA or tRNA genes and all TSS being apart less than 20 bp were excluded. TSS matching the boundaries of RNA-Seq predicted 5’UTRs or ncRNAs were determined accordingly to the flow chart depicted in Additional file 1: Figure S6B.

#### Transcriptome Viewer

Additionally, the gained RNA-Seq data were used to generate logarithmic scaled, color coded graphs representing strand-specific transcription.

### Operon prediction

Operon predictions based on whole transcriptome sequencing, dRNA-Seq transcription start sites, and operon and transcription terminator site determination with DOOR [72], OperonDB [73], and TransTermHP [74]. Operon predictions were curated manually as described by Sharma et al. [32], regarding especially level shifts in transcriptional activity.

### Reannotation

Functional reannotation was carried out using the ERGO software tool (Integrated Genomics, Chicago, USA) [75] and the IMG/ER (Integrated Microbial Genomes/Expert Review) system [45]. Subsequent manual curation was based on the results of a bidirectional BLAST analysis comprising *B. subtilis*, *B. pumilus* and related, manually annotated organisms, the comparisons to UniProtKB/Swiss-Prot and UniProtKB/TrEMBL databases [71] and the analysis of functional domains with InterProScan [70]. The annotation of new genes and the correction of reading frames was based on transcriptional activity and was performed upon analysis of GC frame plots, ribosome-binding sites and -10 and -35 promoter regions using Artemis v12 [76] and comparisons to UniProtKB/Swiss-Prot, UniProtKB/TrEMBL, and InterProScan [70,71]. The removal of gene annotations relied on the combined evaluation of GC frame plots, ribosome-binding sites and -10 and -35 promoter regions using Artemis v12 [76] and comparisons to UniProtKB/Swiss-Prot, UniProtKB/TrEMBL, and InterProScan [70,71]. The absence of transcriptional activity was not used to support the removal of gene annotations. Prophage regions have been annotated by an initial bioinformatic search using Prophagefinder [77] followed by manual evaluation of the candidate regions. Based on the existence of GC content deviations, genes in these regions with significant similarities to known prophages and the identification of insertion repeats, genomic regions were assigned as prophages. The annotation followed the principles of prophage annotation outlined by Casjens [78]. The reannotated data set has been used to update the *B. licheniformis* DSM13 genome data initially submitted by Veith et al. [1] and is now available at NCBI under accession number AE017333.1.

### Clustering of ncRNAs

Cluster analysis to elucidate the fundamental types of ncRNA expression profiles was performed based on the respective NPKM values (Additional file 2: Table S2). To ensure that the data of each replicate are sufficiently reliable, t-tests were performed with MeV [79]. For at least three out of the five samples, the respective ncRNA had to have a P value <0.15 to be taken into further analysis, as described by Koburger et al. [80]. Furthermore, all ncRNAs taken into analysis had to have a minimal NPKM value >10. Means of the replicates of each sampling point were built and z-score transformation was performed. The number of clusters was determined by *Figure of merit* (FOM) analysis, which basically is an estimate of the predictive power of a clustering algorithm [81]. Clusters were generated by employing *k*-means clustering [47] with Euclidian distances in the MeV software [79] and subsequent manual curation.

### Utilized software and databases

#### ACT and Mauve

The comparison of RNA features from *B. licheniformis* with the reference genome *B. subtilis* was based on sequence similarity analyzed with ACT v11, the Artemis comparison tool [82]. Quantification of ncRNAs located in conserved or not-conserved loci, was done employing the progressive Mauve alignment tool [83].

#### baySeq

Determination of constitutive or differential expression of the RNA features was employed with baySeq [49], which uses an empirical Bayes approach assuming a negative binomial distribution and is capable of dealing with multi-group experimental designs. Input data were generated by counting the reads referring to every gene.

#### DOOR and OperonDB

Predictions for operons were thankfully downloaded from the DOOR Database of prOkaryotic OpeRons [72] and OperonDB [73].

#### Gem mappability

The determination of the genome mappability was calculated for a read length of 50 nt with the Gem mappability program [84].

#### MeV

Cluster analysis was performed using the Multiexperiment Viewer v4.8 [79].

#### Rfam

Annotation of *cis*-regulatory elements and small RNAs was carried out by Infernal searches [85] of RNA features versus the Rfam database [46].

#### TransTermHP

Transcription terminators pre-computed with TransTermHP v2.07 were gratefully downloaded from transterm.cbcb.umd.edu [74]. 3’UTRs were checked for terminators as described by Martin et al. [86]. Terminators were considered as internal if they were located at least 50 nt upstream of the end of the transcript.

### Northern blot analysis

*B. licheniformis* DSM13 was cultivated at 37°C and 160 rpm in a 5 L Erlenmeyer flask on defined minimal medium [87]. Cells were harvested at OD_600_ 1 and 4.5 and after having reached the stationary phase for at least 2 h. *Escherichia coli* DH5α was cultivated in Luria broth at 37°C and 180 rpm to an OD_600_ of 2. RNA was isolated as described in *RNA isolation and preparation*. Digoxigenin-labeled RNA probes were prepared by *in vitro* transcription with T7 RNA polymerase (DIG Northern Starter Kit, Roche). Templates for *in vitro* transcription were generated by PCR using primer pairs (Additional file 2: Table S17) containing a primer flanked with the T7 promoter sequence. Gel electrophoresis of the RNA was carried out using a 1% agarose formaldehyde MOPS gel [88] with 100 V applied for 2,5 h. RNA was transferred to the membrane (Nylon Membranes, positively charged, Roche) via vacuum blotting with the Amersham VacuGene XL Vacuum Blotting System (GE Healthcare) using the recommended protocol. The RNA probe hybridization pro-cedure was performed following the manufacturer’s instructions (DIG Northern Starter Kit, Roche). Detection was accomplished with ChemoCam Imager (Intes). RiboRuler High Range RNA Ladder (Thermo Scientific) ranging from 200 to 6000 nt was used as RNA marker.

## Abbreviations

°C: Degrees Celsius; μL: Microliter; A: Adenine; asRNA: Antisense RNA; bp: Base pairs; C: Cytosine; cDNA: Complementary DNA; dRNA-Seq: Differential RNA sequencing; g: Gram; g: Gravitational constant; G: Guanine; h: Hours; L: Liter; ln: Natural logarithm; Mb: Megabase pairs; min: Minute; mL: Milliliter; mM: Millimolar; mRNA: Messenger RNA; ncRNA: Non-coding RNA; NPKM: Nucleotide activity per kilobase of exon model per million mapped reads; nt: Nucleotides; OD: Optical density; ORF: Open reading frame; PCR: Polymerase chain reaction; RNA-Seq: RNA sequencing; rpm: Revolutions per minute; rRNA: Ribosomal RNA; T: Thymine; TAP: Tobacco Acid Pyrophosphatase; TEX: Terminator™ 5′-Phosphate-Dependent Exonuclease; tmRNA: Transfer-messenger RNA; tRNA: Transfer RNA; TSS: Transcription start sites; UTR: Untranslated region; V: Volt; v/v: Volume per volume; w/v: Weight per volume.

## Competing interests

The authors declare that they have no competing interests.

## Authors’ contributions

SW performed the experiments, analyzed data and wrote paper, SD developed the analysis tools, RH performed northern blots, JB and SE provided industrial fermentation facilities and performed the fermentation, SV prepared submission of genome and transcriptome data, RD wrote paper and provided research facilities, HL wrote paper, designed research and analyzed data. All authors read and approved the final version of the manuscript.

## Supplementary Material

Additional file 1: Figure S1. Distribution of whole transcriptome sequencing reads. **Figure S2:** Comparative operon prediction. **Figure S3:** Northern blot confirmation of non-coding RNAs. **Figure S4:** Antisense RNAs with putative impact on productivity. **Figure S5:** BsrG/SR4-like loci in *B. licheniformis*. **Figure S6:** Work flow charts. Click here for file

Additional file 2: Table S1. Whole transcriptome sequencing reads. **Table S2:** NPKM values of RNA features. **Table S3:** NPKM values of all genes. **Table S4:** Differential RNA-Seq reads. **Table S5:** Transcription start sites. **Table S6:** Identified RNA features. **Table S7:** Predicted operons. **Table S8:** Corrected genes. **Table S9:** New genes. **Table S10:** Removed genes. **Table S11:** Predicted *cis*-regulatory elements. **Table S12:** Comparison of *cis*-regulatory elements known from *B. subtilis*. **Table S13:** Cluster analysis of ncRNA expression profiles. **Table S14:** Comparison of ncRNAs to *B. subtilis*. **Table S15:** Comparison of small RNAs from *B. subtilis* to identified ncRNAs. **Table S16:** Sequence Read Archive accession. **Table S17:** Primer pairs for Northern blots. Click here for file
