# Supplementary material for: RNA-Seq of Bacillus licheniformis: active regulatory RNA features expressed within a productive fermentation
Source: BMC Genomics. 2013 Oct 1;14:667. doi: 10.1186/1471-2164-14-667 (PMC3871023; doi:10.1186/1471-2164-14-667)
Supplement: Additional file 1: Figure S1. — Distribution of whole transcriptome sequencing reads. Figure S2: Comparative operon prediction. Figure S3: Northern blot confirmation of non-coding RNAs. Figure S4: Antisense RNAs with putative impact on productivity. Figure S5: BsrG/SR4-like loci in B. licheniformis. Figure S6: Work flow charts. [file 1471-2164-14-667-S1.pdf]

Additional File 1

RNA-Seq of *Bacillus licheniformis*: active regulatory RNA features expressed within a productive fermentation

Sandra Wiegand, Sascha Dietrich, Robert Hertel, Johannes Bongaerts, Stefan Evers, Sonja Volland , Rolf Daniel and Heiko Liesegang

Content

Figure S1 Distribution of whole transcriptome sequencing reads. 2

Figure S2 Comparative operon prediction. 3

Figure S3 Northern blot confirmation of non-coding RNAs. 4

Figure S4 Antisense RNAs with putative impact on productivity. 13

Figure S5 BsrG/SR4-like loci in *B. licheniformis*. 15

Figure S6 Work flow charts. 16

References 17

Figure S1

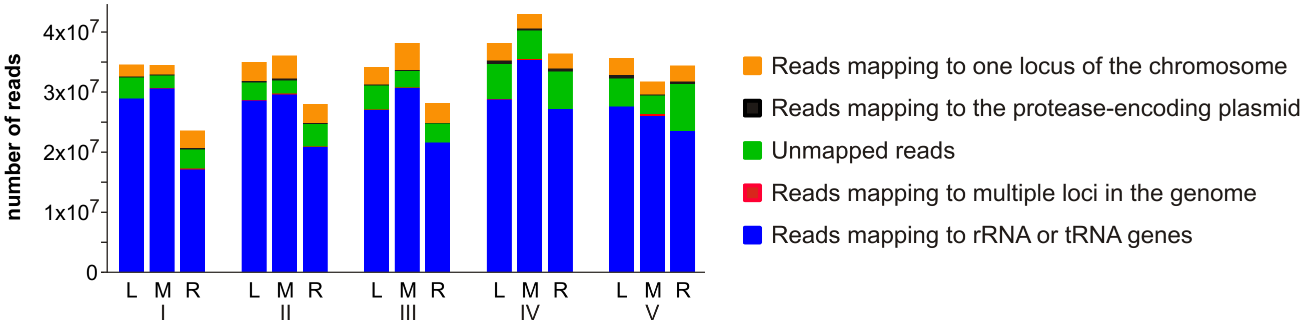

Figure S1 Distribution of whole transcriptome sequencing reads. Mapping results of the RNA-Seq reads to the B. licheniformis genome based on sequence similarity. Results are shown for replicates of the sampling points I to V. The category *unmapped reads* mainly comprises reads derived from experimental artifacts like poly(A) tails or concatenated RNA adapters, as well as reads with more than one sequencing error per 50 bp. Please note that not a single read which has been tested from this fraction can be assigned to other organisms than *B. licheniformis*.

Figure S2

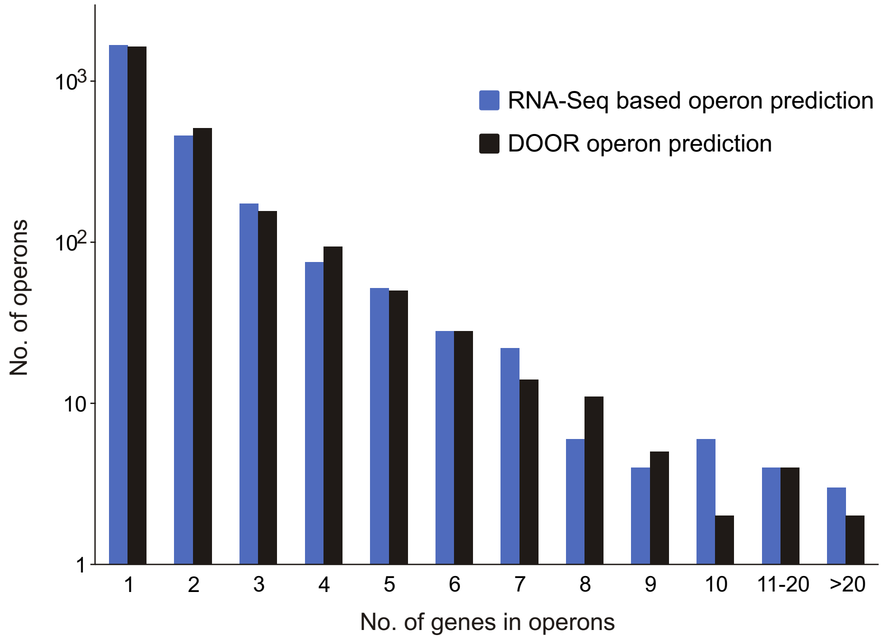

Figure S2 Comparative operon prediction. Number of genes in predicted operons are shown for the *in silico* prediction available at DOOR [1] (black) and the manually curated prediction based on RNA-Seq provided in this study (blue). The deviations especially in the longer operons are due to the here employed expression profile-accounting method of operon prediction. Please note that results are given on log-transformed scale.

Figure S3A

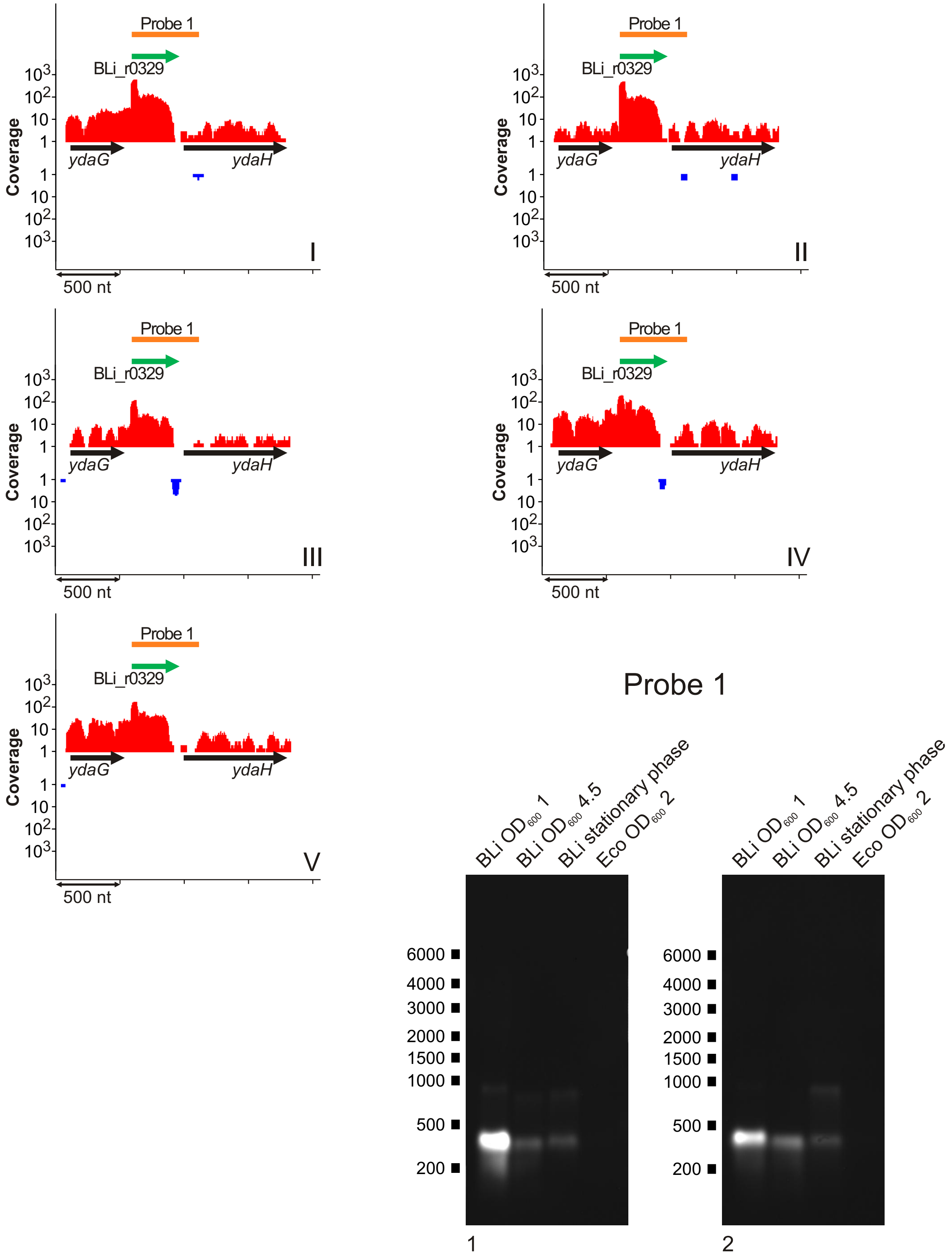

Probe 1
BLi_r0329
ydaG
ydaH
Coverage
I
II
III
IV
V
500 nt
Probe 1
BLi OD600 1
BLi OD600 4.5
BLi stationary phase
Eco OD600 2
6000
4000
3000
2000
1500
1000
500
200
1
2

Figure S3B.1

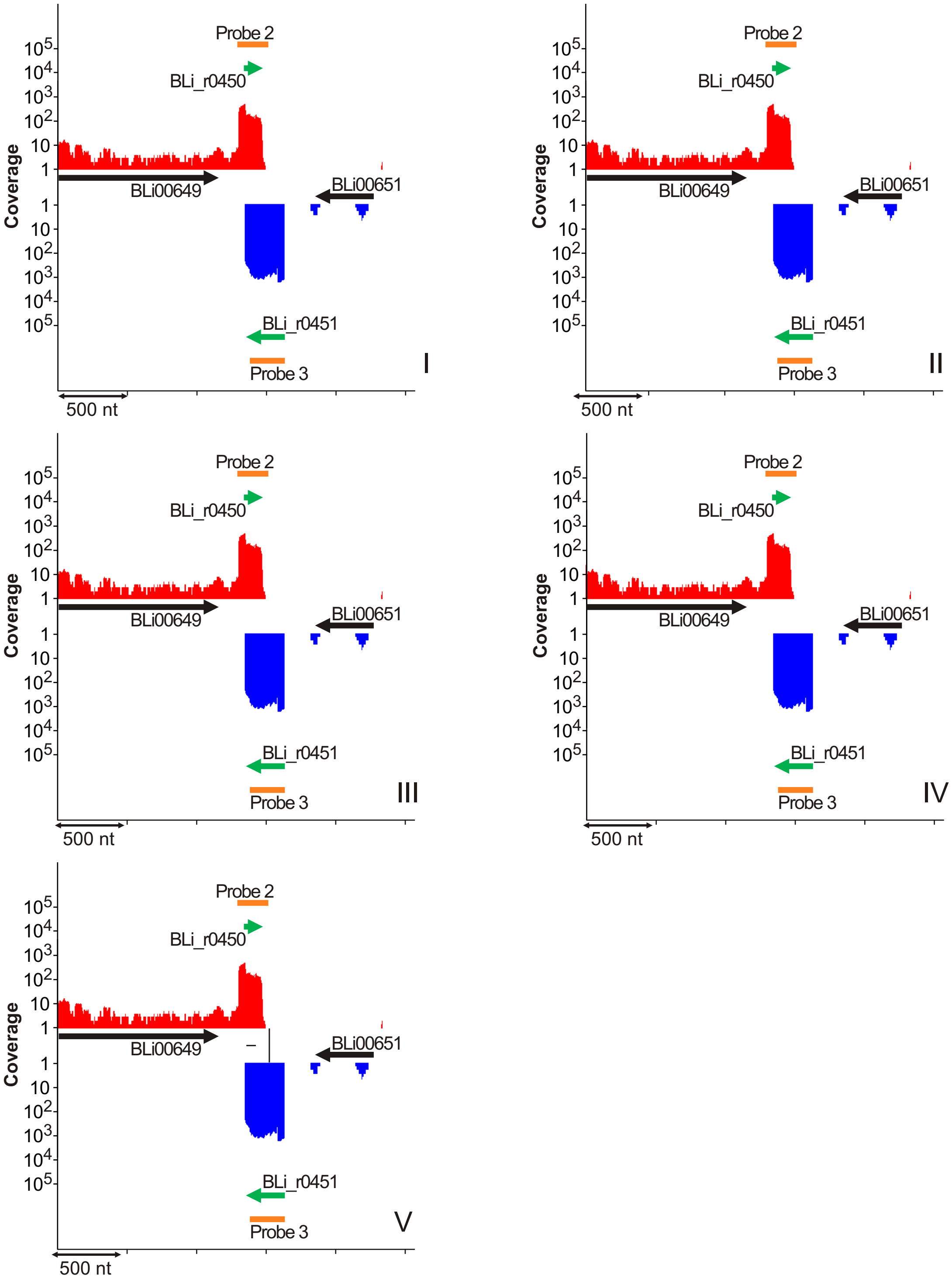
Probe 2
BLi_r0450
BLi00649
BLi00651
BLi_r0451
Probe 3
Coverage
10^5
10^4
10^3
10^2
10
1
500 nt
I
II
III
IV
V

Figure S3B.2

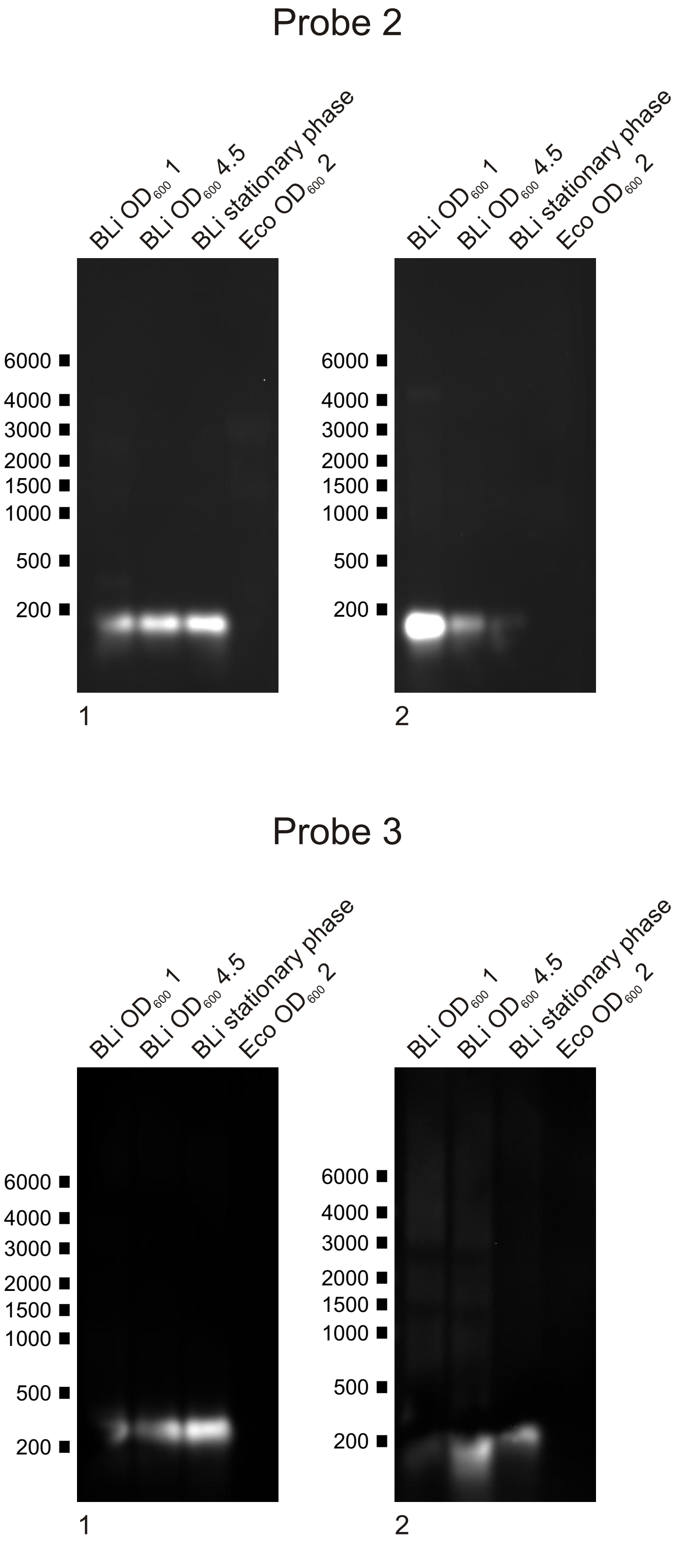
Probe 2
BLi OD600 1
BLi OD600 4.5
BLi stationary phase
Eco OD600 2
6000
4000
3000
2000
1500
1000
500
200
1
2
Probe 3
1
2

Figure S3C

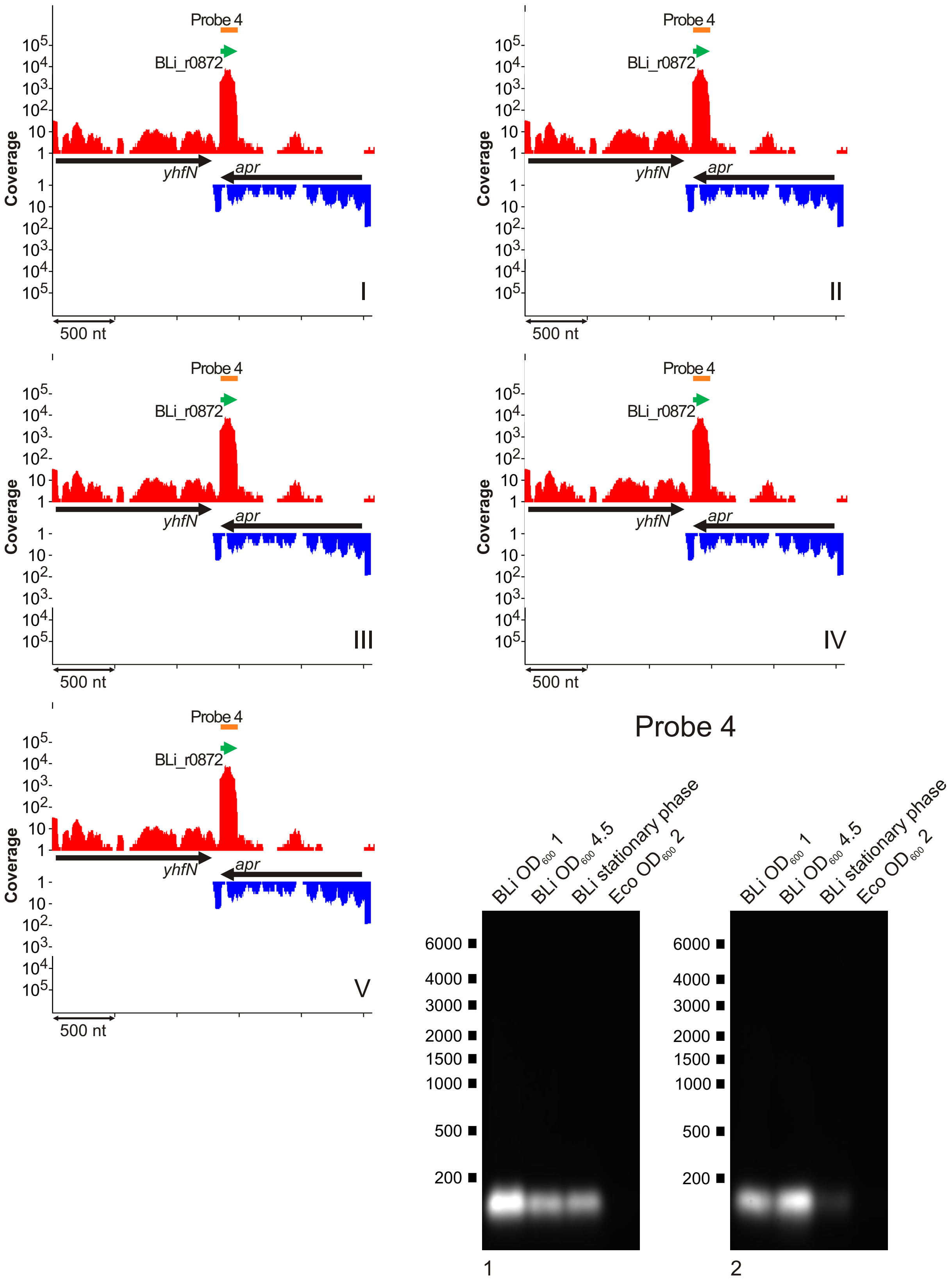
Probe 4
BLi_r0872
Coverage
yhfN
apr
500 nt
I
II
III
IV
V
Probe 4
BLi OD600 1
BLi OD600 4.5
BLi stationary phase
Eco OD600 2
6000
4000
3000
2000
1500
1000
500
200
1
2

Figure S3D

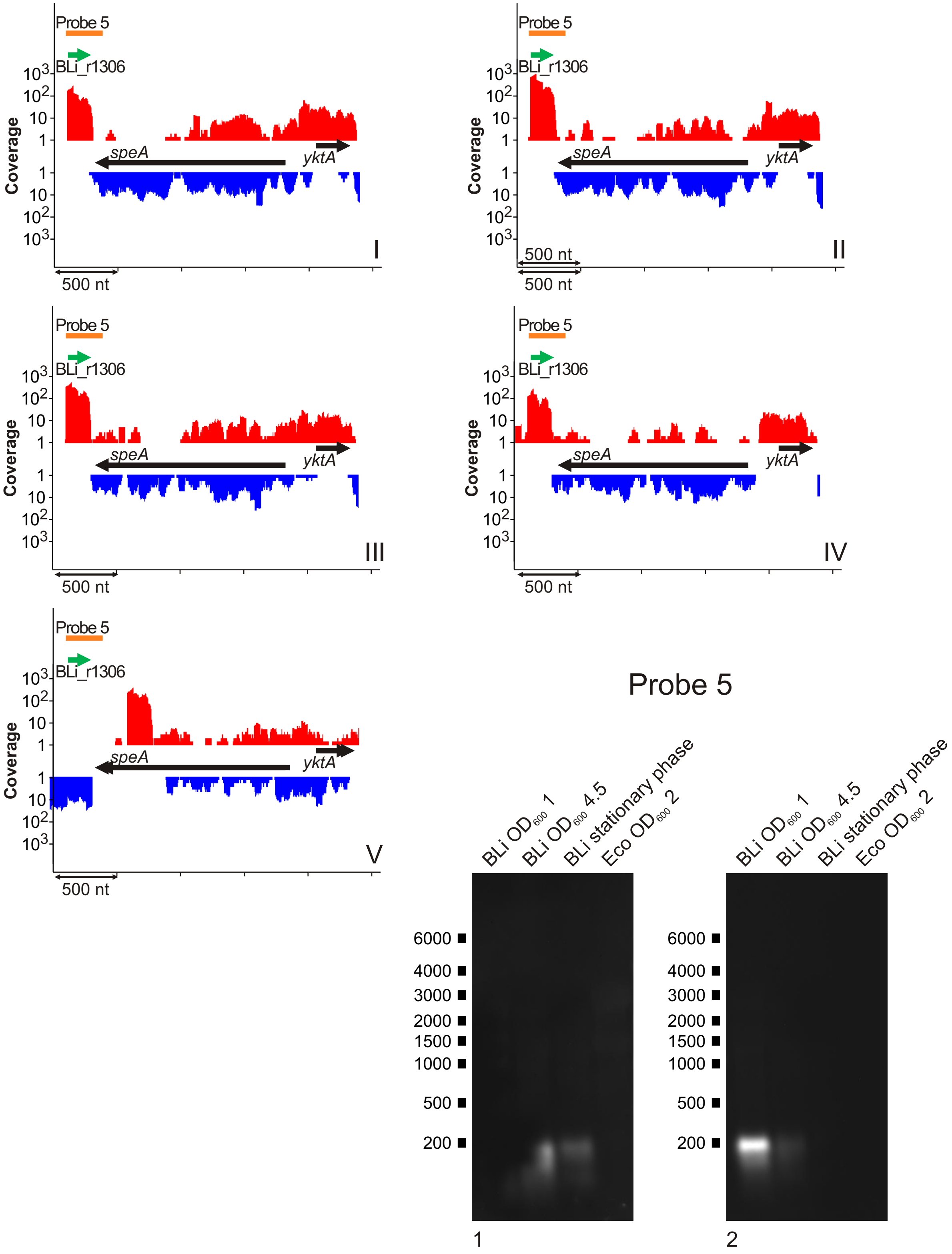
Probe 5
BLi_r1306
Coverage
speA
yktA
500 nt
I
II
III
IV
V
Probe 5
BLi OD600 1
BLi OD600 4.5
BLi stationary phase
Eco OD600 2
6000
4000
3000
2000
1500
1000
500
200
1
2

Figure S3E

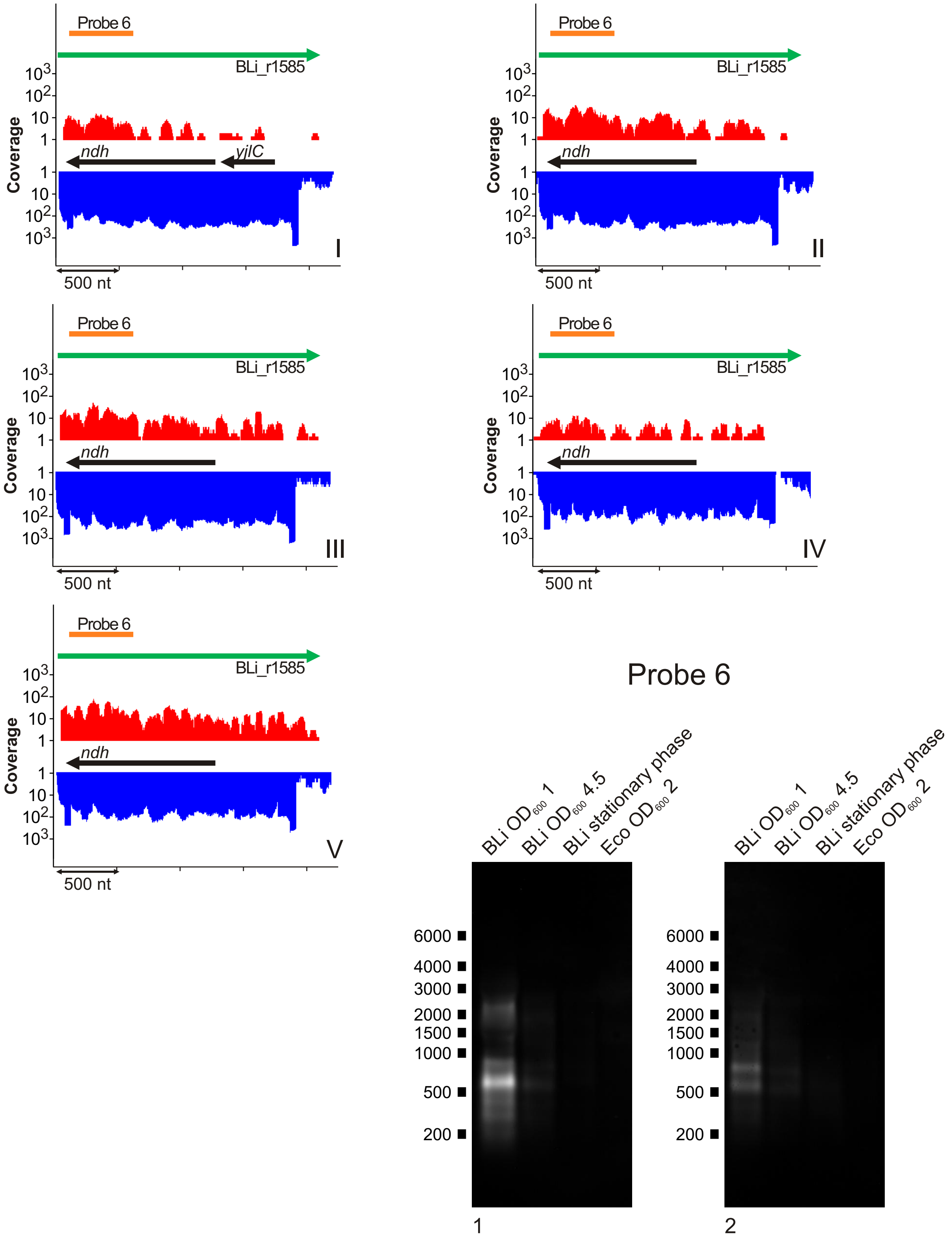

Probe 6
BLi_r1585
Coverage
ndh
yjlC
500 nt
I
II
III
IV
V
Probe 6
BLi OD600 1
BLi OD600 4.5
BLi stationary phase
Eco OD600 2
6000
4000
3000
2000
1500
1000
500
200
1
2

Figure S3F

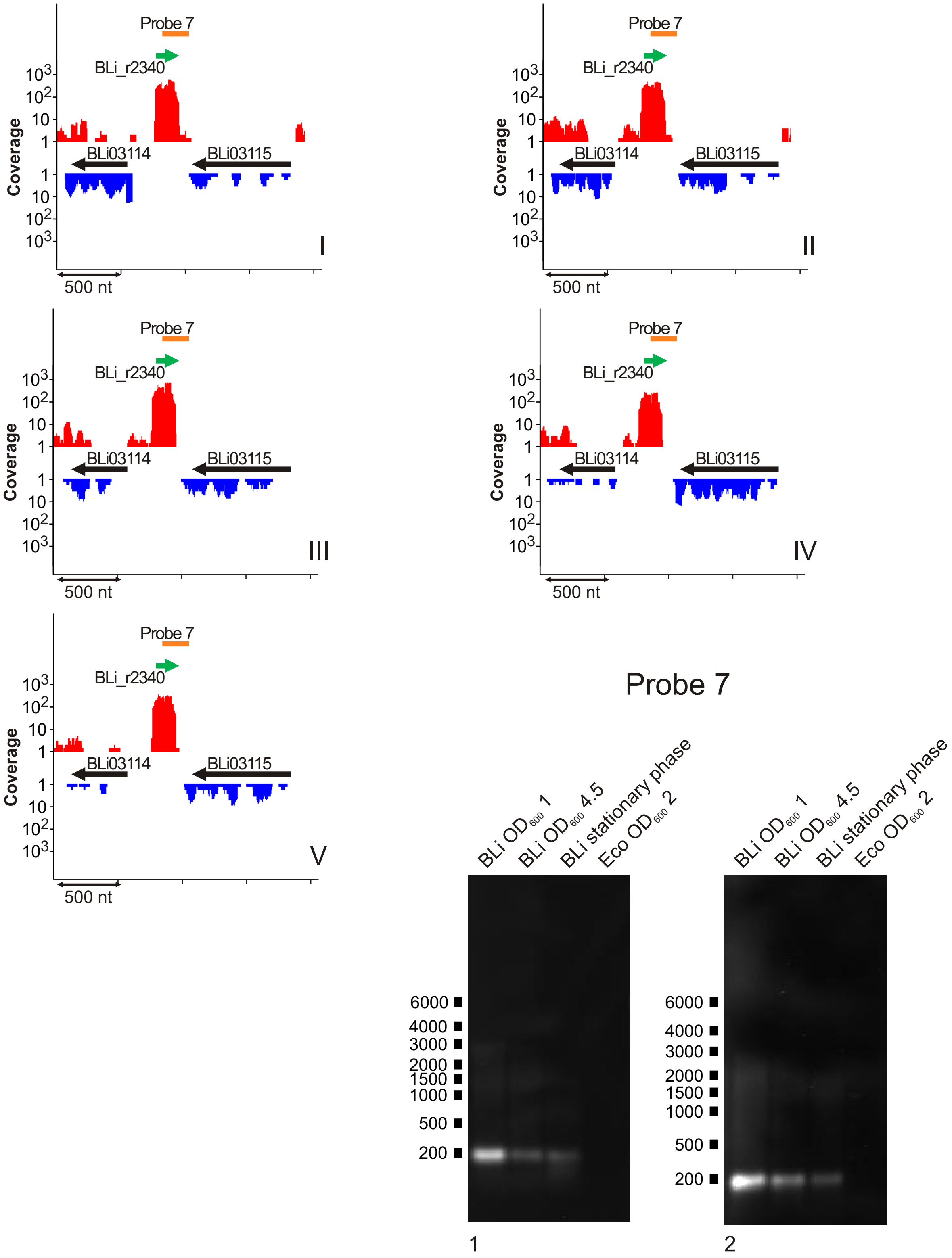
Probe 7
BLi_r2340
BLi03114
BLi03115
Coverage
500 nt
I
II
III
IV
V
Probe 7
BLi OD600 1
BLi OD600 4.5
BLi stationary phase
Eco OD600 2
6000
4000
3000
2000
1500
1000
500
200
1
2

Figure S3G

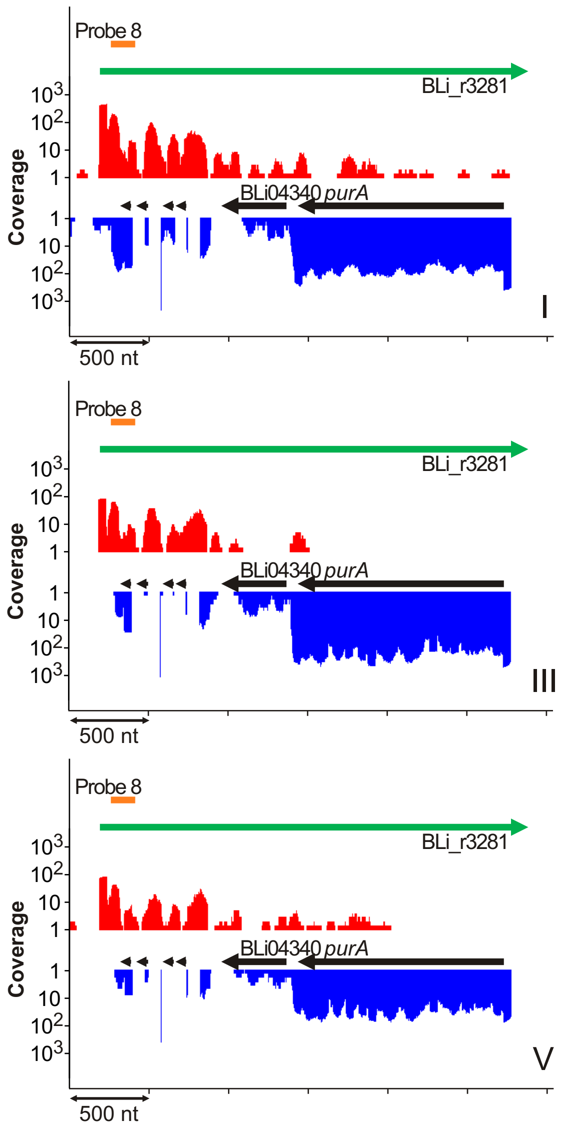

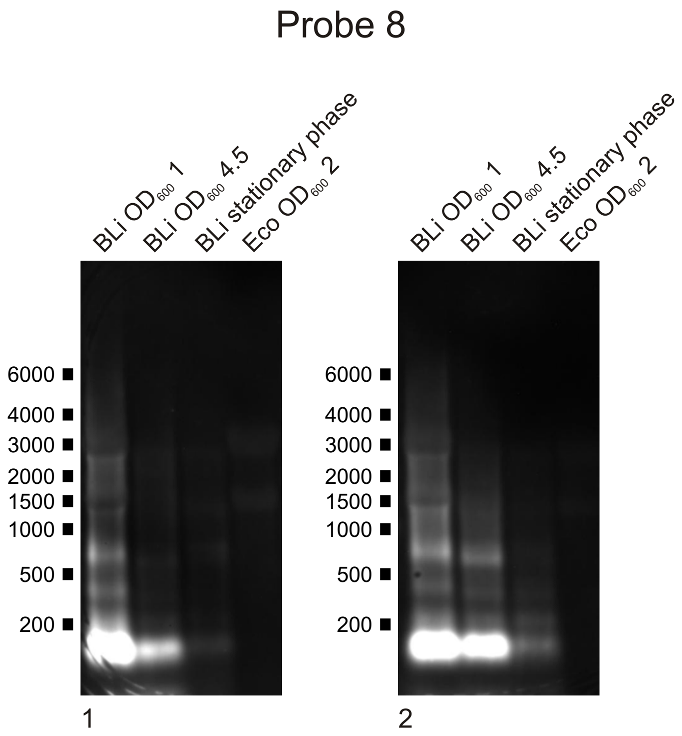

Non-labeled arrows from left to right: *trnF3*, *trnD4*, *trnE6* and *trnK3*. The gaps in coverage at the same position result from removal of reads according to tRNA genes before mapping.

Figure S3 Northern blot confirmation of non-coding RNAs. For each blot the corresponding genomic loci and the transcriptional activity is shown for all sampling points. The black arrows show genes, the green arrow the respective ncRNA. Probes (Additional File 2: Table S17) are marked orange. Northern blotting was carried out as described in *Methods*. The size range is given in base pairs and the number under each picture signifies the different replicates. **(A)** *indep* RNA BLi_r0329 (expected size: 373 bp). **(B)** *indep* RNAs BLi_r0450 and BLi_r0451 (expected size: 127 bp and 291 bp, respectively). **(C)** A_I RNA BLi_r0872 (expected size: 144 bp). This ncRNA is of special interest since it is encoded antisense to the alkaline protease Subtilisin Carlsberg. **(D)** A_{misc} RNA BLi_r1306 (expected size: 205 bp). Please note that within the same transcript an ORF was annotated "SR1-like protein", according to the finding in *B. subtilis,* where SR1 acts as functional sRNA and encodes a protein. **(E)** A_{misc} RNA BLi_r1585 (expected size: 2072 bp). The observed bands indicate processing or degradation of the RNA transcript. **(F)** *indep* RNA BLi_r2340 (expected size: 188 bp) **(G)** A_{misc} RNA BLi_r3281 (expected size: 2688 bp). The observed bands may indicate a fading-out of the transcription after leaky termination.

Figure S4A-D

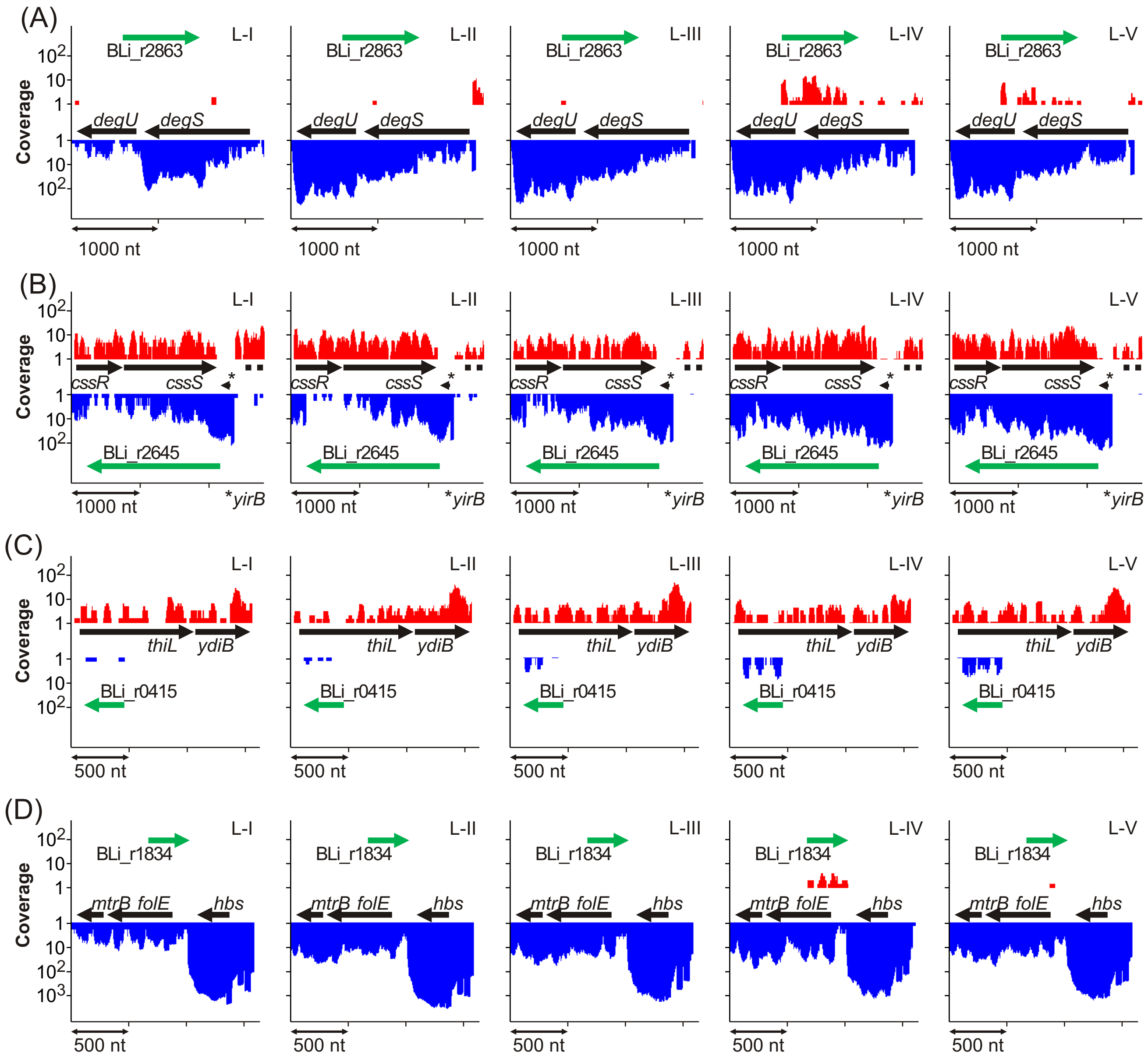
(A)
L-I
L-II
L-III
L-IV
L-V
BLi_r2863
degU
degS
Coverage
10^2
10
1
1
10
10^2
1000 nt
(B)
cssR
cssS
*
BLi_r2645
*yirB
(C)
thiL
ydiB
BLi_r0415
500 nt
(D)
BLi_r1834
mtrB
folE
hbs
10^3

Figure S4E-G

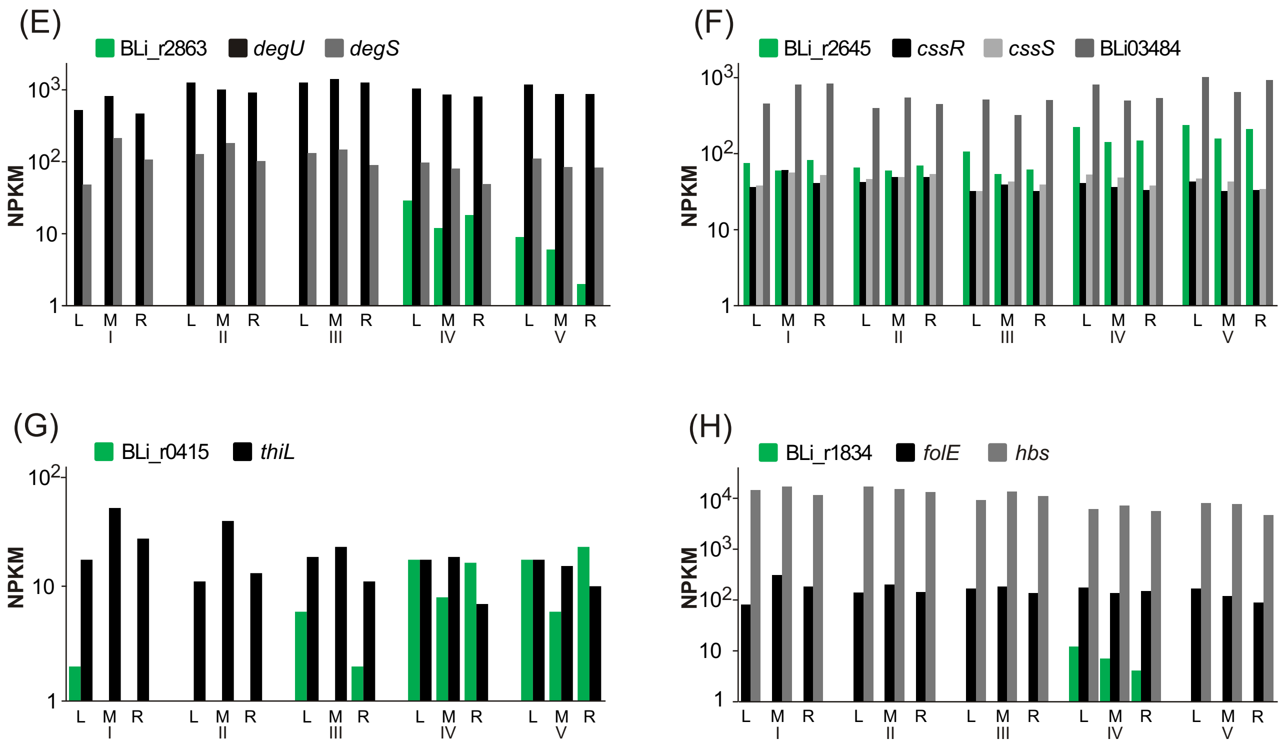

Figure S4 Antisense RNAs with putative impact on productivity. Antisense transcriptional activities at different fermentation stages. Black arrows indicate genes and green arrows identify antisense RNAs. **(A)** A_{misc} RNA BLi_r2863, **(B)** $A_{3'UTR}$ BLi_r2645, **(C)** A_{misc} RNA BLi_r0415 and **(D)** A_{misc} BLi_r1834 are shown for L-I to L-V. NPKM values are displayed for all replicates of the sampling points I to V for: **(E)** BLi_r2863 and the *degSU* operon, a two-component regulatory system which is involved in the manifestation of multicellular communities [2] and regulates biofilm formation, genetic competence, swarming motility, polyglutamic acid production as well as exoprotease secretion [3, 4]. As the transcript is present in the productive stages of the fermentation process, an influence on protease secretion via an impact on the *degSU* mRNA should be carefully considered. In *B. subtilis*, an ncRNA antisense to *degSU* is also annotated [5], but varies in length and start position due to a disparate genomic context. **(F)** BLi_r2645 and the *cssRS* operon, a two-component regulatory system in control of the serine proteases HtrA and HtrB [6], which bear proteolytical as well as chaperone activity in response to secretion stress [7]. The overlapping 3'UTR antisense to the *cssRS* operon is generated by the proteolytical anti-adaptor protein YirB. **(G)** BLi_r0415 and *thiL*, which encodes a thiamine-monophosphate kinase; **(H)** BLi_r1834 and *folE*, which encodes a GTP cyclohydrolase I involved in folate biosynthesis (plus *hbs*, the adjacent DNA-binding protein HBsu). Please note that results are given on log-transformed scale.

Figure S5

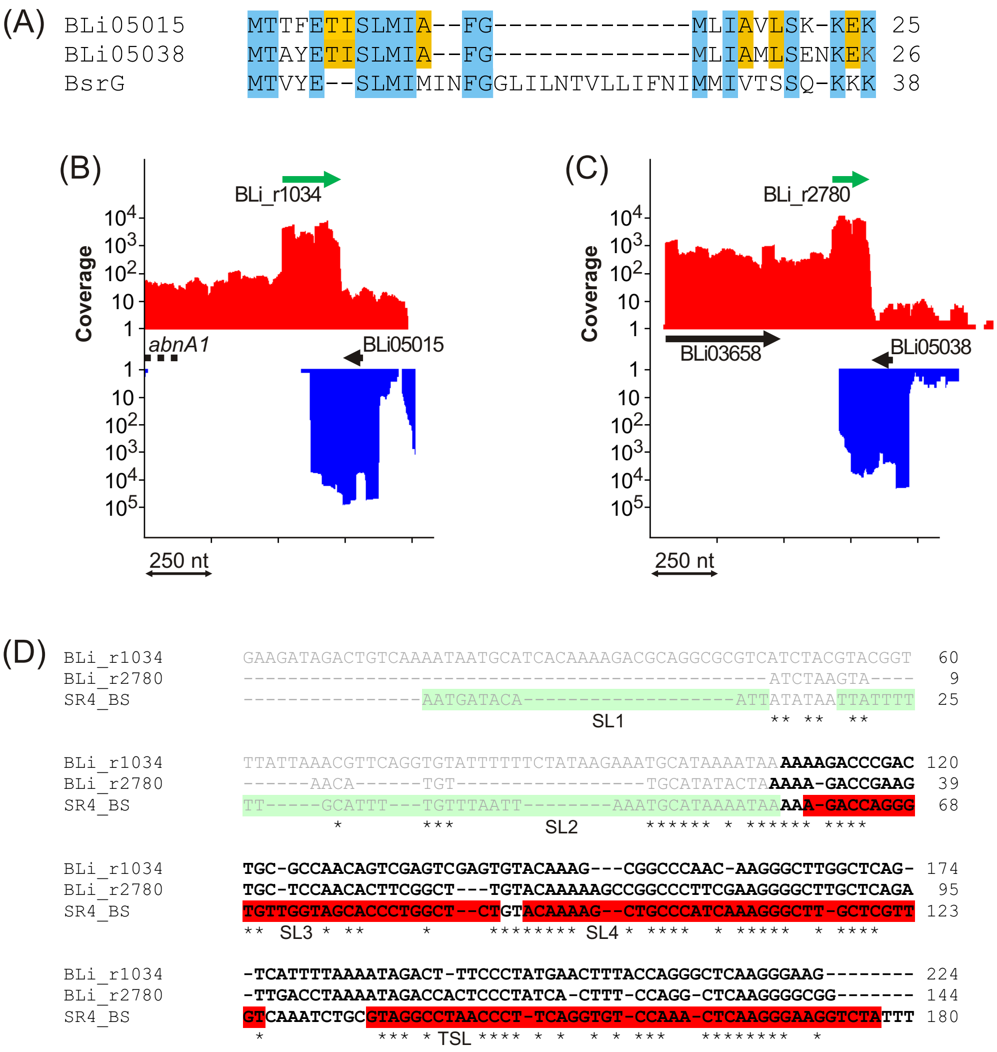

Figure S5 BsrG/SR4-like loci in *B. licheniformis*. (A) ClustalW2 alignment of BsrG from *B. subtilis* [8] and the peptides deduced from the newly annotated genes BLi05015 and BLi05038. Blue boxes show amino acids conserved in all three peptides and orange boxes amino acids only conserved between the two *B. licheniformis* peptides. **(B)** BLi05015 (1300390-1300316) indicated by the black arrow and BLi_r1034 (green arrow). **(C)** Transcriptional activities of BLi05038 (3485388-3485308) indicated by the black arrow and BLi_r2780 (green arrow). **(D)** ClustalW2 alignment of SR4 from *B. subtilis* [8] and *indep* RNAs BLi_t1034 and BLi_r2780. Nucleotides overlapping the opposite mRNA are pictured black and bold, non-overlapping nucleotides are marked gray. The green and red boxes indicate the stem loops SL1 to SL4 and TSL identified for SR4 [8] in the non-overlapping and the overlapping region, respectively.

Figure S6

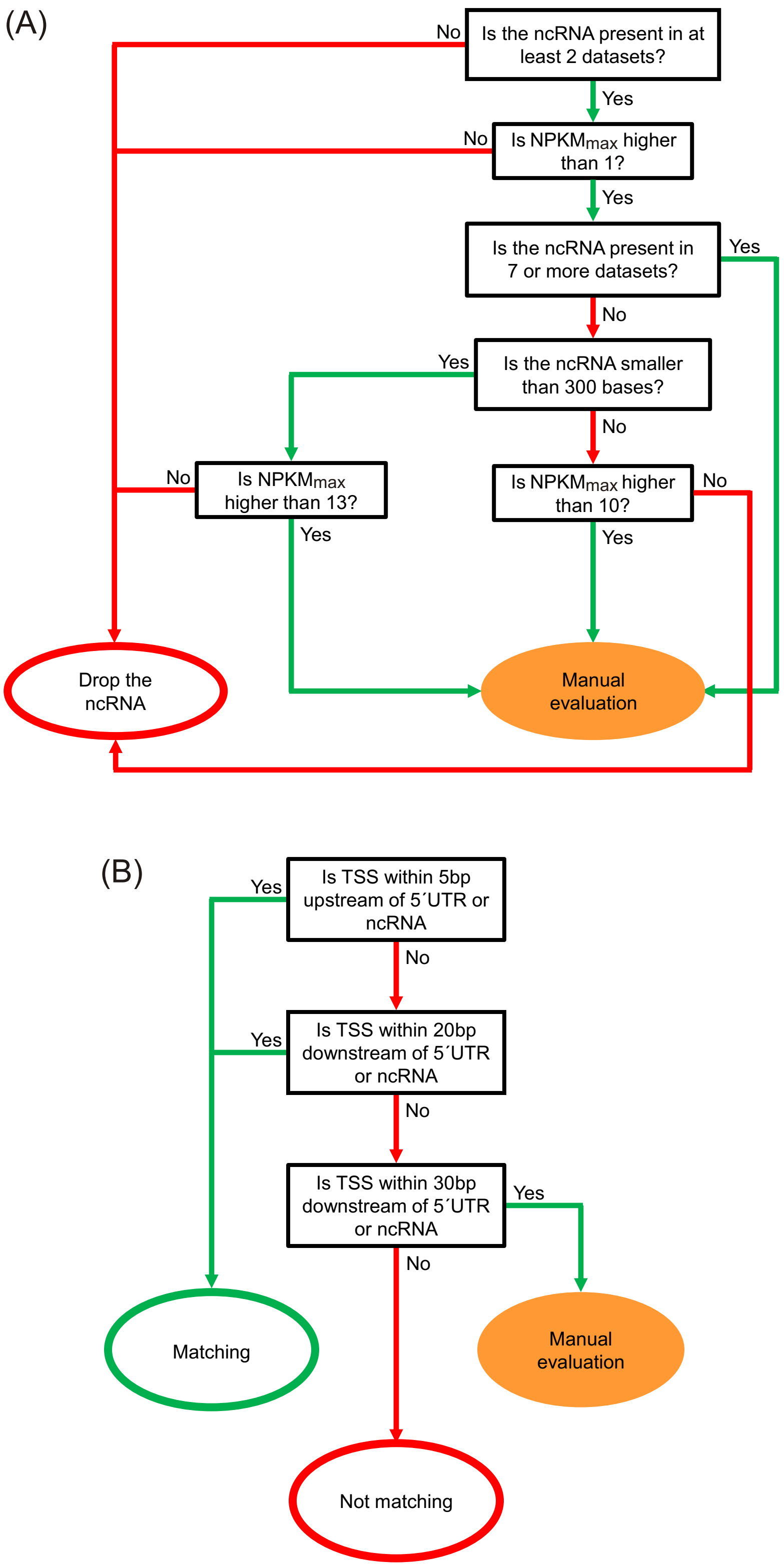

Figure S6 Work flow charts. (A) Work flow of non-coding RNA evaluation. **(B)** Work flow of transcription start site evaluation.

References

1. Mao F, Dam P, Chou J, Olman V, Xu Y: **DOOR: a database for prokaryotic operons.** *Nucleic Acids Res* 2009, **37**:D459–63.

2. Murray EJ, Kiley TB, Stanley-Wall NR: **A pivotal role for the response regulator DegU in controlling multicellular behaviour.** *Microbiology+* 2009, **155**:1–8.

3. Hoffmann K, Wollherr A, Larsen M, Rachinger M, Liesegang H, Ehrenreich A, Meinhardt F: **Facilitation of direct conditional knockout of essential genes in *Bacillus licheniformis* DSM13 by comparative genetic analysis and manipulation of genetic competence.** *Appl Environ Microbiol* 2010, **76**:5046–57.

4. Davidson F, Seon-Yi C, Stanley-Wall N: **Selective Heterogeneity in Exoprotease Production by *Bacillus subtilis*.** *PLoS One* 2012, **7**:e38574.

5. Nicolas P, Mäder U, Dervyn E, Rochat T, Leduc A, Pigeonneau N, Bidnenko E, Marchadier E, Hoebeke M, Aymerich S, Becher D, Bisicchia P, Botella E, Delumeau O, Doherty G, Denham EL, Fogg MJ, Fromion V, Goelzer A, Hansen A, Hartig E, Harwood CR, Homuth G, Jarmer H, Jules M, Klipp E, Le Chat L, Lecointe F, Lewis P, Liebermeister W, et al.: **Condition-Dependent Transcriptome Reveals High-Level Regulatory Architecture in *Bacillus subtilis*.** *Science* 2012, **335**:1103–1106.

6. Darmon E, Noone D, Masson A, Bron S, Kuipers OP, Devine KM, van Dijl JM: **A Novel Class of Heat and Secretion Stress-Responsive Genes Is Controlled by the Autoregulated CssRS Two-Component System of *Bacillus subtilis*.** *J Bacteriol* 2002, **184**:5661.

7. Marciniak BC, Trip H, van-der Veek PJ, Kuipers OP: **Comparative transcriptional analysis of *Bacillus subtilis* cells overproducing either secreted proteins, lipoproteins or membrane proteins.** *Microb Cell Fact* 2012, **11**:66.

8. Jahn N, Preis H, Wiedemann C, Brantl S: **BsrG/SR4 from *Bacillus subtilis*--the first temperature-dependent type I toxin-antitoxin system.** *Mol Microbiol* 2012, **83**:579–98.
